# USP11‐PGAM5 Axis Promotes Neurotoxic Astrocyte Reactivity by Aggravating the mtDNA‐cGAS‐STING Pathway After Intracerebral Hemorrhage

**DOI:** 10.1002/advs.202514283

**Published:** 2025-11-07

**Authors:** Jiaqing He, Zijuan Qin, Yaning Cai, Haixiao Liu, Tinghao Wang, Qing Hu, Yanni Xu, Pan Yang, Xun Wu, Yan Qu, Wei Guo

**Affiliations:** ^1^ Department of Neurosurgery Xi'an Medical University Xi'an Shaanxi 710021 China; ^2^ Department of Neurosurgery Tangdu Hospital the Fourth Military Medical University Xi'an Shaanxi 710032 China; ^3^ School of Medicine Northwest University 229 Taibai North Road Xi'an 710069 China; ^4^ Department of Biomedical Engineering Fourth Military Medical University Xi'an Shaanxi 710032 China; ^5^ Department of Neurosurgery The 83rd Affiliated Hospital of Xinxiang Medical University Xinxiang Henan China; ^6^ Department of Neurosurgery First Affiliated Hospital of Gannan Medical University Ganzhou Jiangxi 341000 China; ^7^ Department of Emergency Tangdu Hospital Fourth Military Medical University Xi'an Shaanxi 710032 China

**Keywords:** cGAS‐STING, Intracerebral hemorrhage, Neurotoxic astrocytes, PGAM5, USP11

## Abstract

Astrocyte transformation into a neurotoxic phenotype is a hallmark pathological feature of intracerebral hemorrhage (ICH) and a key contributor to exacerbating secondary brain injury. Understanding the underlying mechanisms that regulate the reactivity of neurotoxic astrocytes is critical for developing targeted treatment strategies for ICH. In this study, analyses of perihematomal tissue from ICH patients and mice reveal that neurotoxic astrocyte reactivity correlates with expression levels of phosphoglycerate mutase family member 5 (PGAM5). Furthermore, USP11 is identified as a novel deubiquitinase of PGAM5, which contributes to elevated PGAM5 expression in neurotoxic astrocytes by suppressing its ubiquitin‐proteasome degradation. Astrocyte‐specific *Usp11* or *Pgam5* knockout suppresses neurotoxic astrocyte reactivity, reduces neuronal apoptosis, and facilitates neurofunctional recovery. Mechanistically, PGAM5 promotes mitochondrial permeability transition pore (mPTP) opening and dynamin‐related protein 1 (Drp1) mediated mitochondrial fission, synergistically amplifying mitochondrial DNA (mtDNA) leakage, and cGAS‐STING pathway activation. Clearing mtDNA or blocking cGAS‐STING pathway effectively attenuates USP11‐PGAM5‐driven neurotoxic astrocyte reactivity. Furthermore, extracellular vesicles modified by angiopep‐2 are engineered for blood‐brain barrier‐penetrating delivery of *Pgam5*‐targeting small interfering RNA, which significantly improves neurological function after ICH. In summary, the findings unveil an unrecognized mechanistic role for the USP11‐PGAM5 axis in driving neurotoxic astrocyte reactivity by regulating the mtDNA‐cGAS‐STING pathway after ICH.

## Graphical abstract

This study reveals that the deubiquitinase USP11 stabilizes PGAM5, triggering neurotoxic astrocyte transformation after intracerebral hemorrhage. PGAM5 promotes mPTP opening and Drp1 dephosphorylation, synergistically amplifying mtDNA leakage into the cytosol and leading to cGAS–STING hyperactivation. Genetic knockout or pharmacological inhibition of *Pgam5* attenuates brain injury and enhances neurological functional recovery, offering a novel therapeutic strategy.

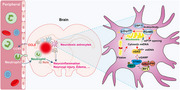

## 1 Introduction

Intracerebral hemorrhage (ICH) is a life‐threatening form of acute brain injury, resulting in ≈2.8 million deaths worldwide annually.^[^ ^1^ ^]^ Due to the irreversible neurological damage and inadequate treatment options, survivors of ICH often suffer from neurological function deficits. Astrocytes, the most abundant resident cells in the central nervous system (CNS), play a crucial role in maintaining CNS homeostasis and performing various complex functions.^[^ ^2^ ^]^ In response to ICH injury, astrocytes undergo a process known as “reactive astrocytosis,” which has emerged as a pathological hallmark of ICH.^[^ ^3^, ^4^ ^]^ Recent studies have revealed that reactive astrocytes exhibit marked heterogeneity and can exert both detrimental and neuroprotective effects within the CNS.^[^ ^5^, ^6^ ^]^ Among these, neurotoxic astrocytes have been reported to be induced by inflammatory microglia through their secretion of IL‐1α, TNF‐α, and C1q.^[^ ^7^, ^8^ ^]^ This phenotype is characterized by the loss of essential astrocytic functions and increased release of neurotoxins.^[^ ^7^ ^]^ Furthermore, such astrocytes can exacerbate brain injury by remodeling the cerebral immune microenvironment via secretion of proinflammatory factors and chemokines. Although previous studies have established that microglia‐derived IL‐1α, TNF‐α, and C1q act as extrinsic drivers of astrocyte neurotoxicity, the intrinsic molecular pathways governing neurotoxic astrocytes remain poorly understood, and targeted strategies to modulate this phenotype are lacking.

Mitochondria are crucial for both integrating and releasing information.^[^ ^9^ ^]^ Upon experiencing damage, mitochondria unleash damage‐associated molecular patterns (DAMPs),^[^ ^10^, ^11^ ^]^ including reactive oxygen species (ROS),^[^ ^12^ ^]^ calcium,^[^ ^13^ ^]^ cytochrome c,^[^ ^14^ ^]^ and other unidentified signals. More recently, the leakage of mitochondrial DNA (mtDNA) into the cytosol has emerged as a significant trigger of inflammatory response,^[^ ^15^, ^16^, ^17^ ^]^ potentially through the activation of inflammatory pathways such as cGAS‐STING.^[^ ^18^, ^19^ ^]^ Nonetheless, the precise contribution of abnormal mtDNA homeostasis to neurotoxic astrocyte reactivity remains enigmatic.

Phosphoglycerate mutase family member 5 (PGAM5), a mitochondrial membrane‐bound phosphatase, is a critical regulator of mitochondrial dynamics and cell death.^[^ ^20^, ^21^, ^22^ ^]^ For example, PGAM5 exacerbates podocyte injury and accelerates diabetic kidney disease progression by promoting mitochondrial fragmentation through dephosphorylation of dynamin‐related protein 1 (Drp1).^[^ ^23^ ^]^ However, the precise involvement of PGAM5 in the reactivity of neurotoxic astrocytes and the progression of ICH remains undisclosed.

In this study, we discovered a correlation between PGAM5 expression and neurotoxic astrocyte reactivity in clinical ICH patients and mouse ICH models. Subsequent animal experiments revealed that the elevation of astrocytic PGAM5 post‐ICH is driven by enhanced ubiquitin‐specific protease 11 (USP11)‐mediated deubiquitination. Specific knockout of either *Pgam5* or *Usp11* significantly ameliorated secondary brain injury. Furthermore, we established that ICH induces neurotoxic astrocytes through USP11‐PGAM5‐mediated mtDNA leakage and cGAS‐STING pathway activation. Moreover, considering the translational potential, we employed extracellular vesicles (EVs)‐mediated delivery of small interfering RNA (siRNA) targeting *Pgam5* (si*Pgam5*) to astrocytes as a therapeutic strategy, demonstrating significant efficacy. Collectively, our findings establish a novel role of the USP11‐PGAM5 axis in neurotoxic astrocytes and secondary brain injury following ICH, and provide potential therapeutic strategies for the clinical translation of ICH.

## 2 Results

### 2.1 PGAM5 Protein was Increased in Neurotoxic Astrocytes Independent of Transcriptional Regulation During the Acute Phase of ICH in Patients and Mice

**Figure 1:**
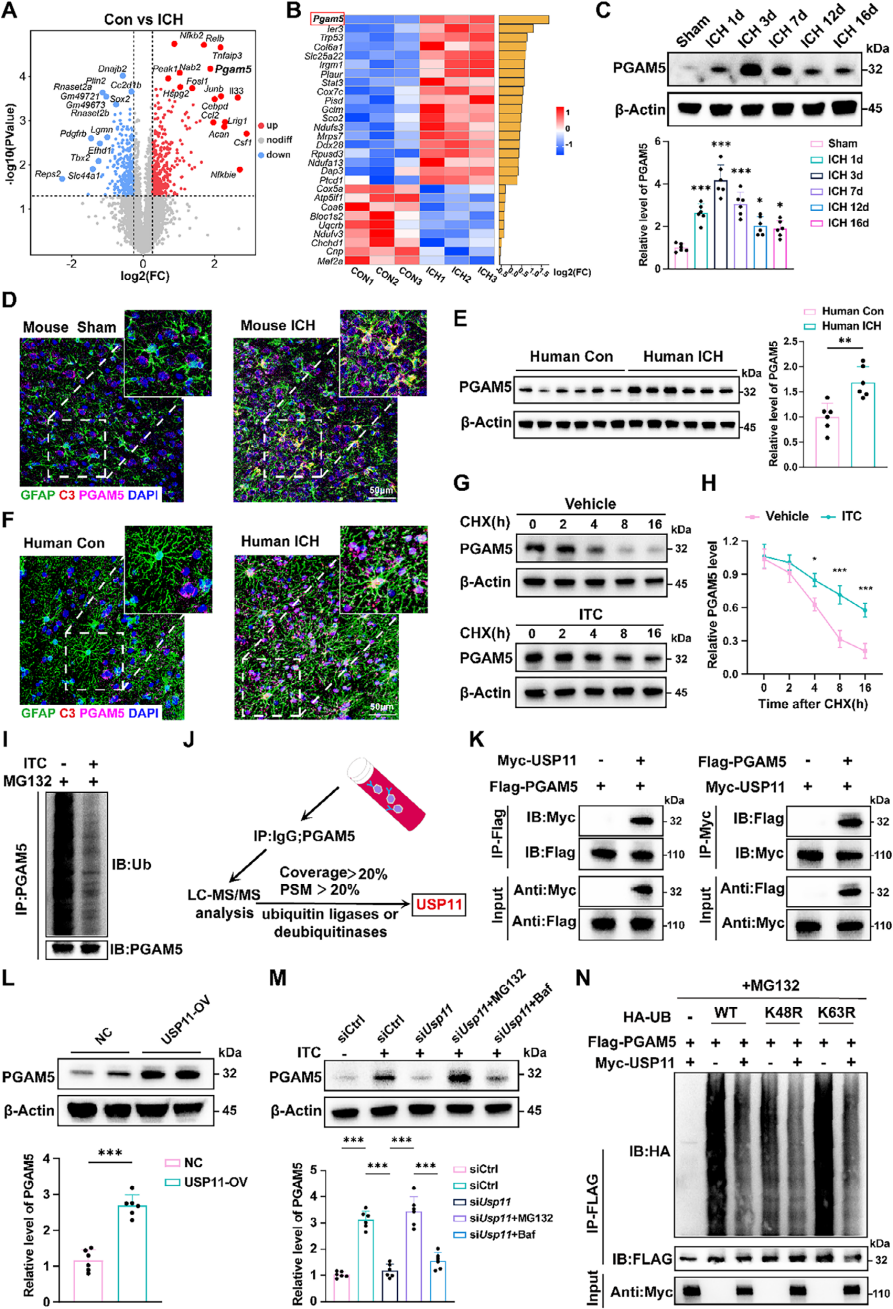
The increase of PGAM5 expression due to USP11 deubiquitinates PGAM5 to suppress its ubiquitin‐proteasome degradation. A) Volcano plot of differentially expressed proteins from proteomic sequencing. B) Enrichment heatmap of mitochondria‐related gene expression. C) Western blot analysis was conducted on PGAM5 in isolated astrocytes at various time intervals after ICH surgery in mice (*n* = 6 per group, one‐way ANOVA). The asterisk at the top indicates the comparison between this group and the Sham group. D) Representative immunofluorescence staining of GFAP, C3, and PGAM5 at three days post‐ICH surgery in mice. Scale bar: 50 µm. E) Analysis of PGAM5 via Western blot in astrocytes extracted from brain samples of individuals with ICH and control groups. (6 samples for ICH group, 6 samples for control group, Student's *t*‐test). F) Representative immunofluorescence staining of GFAP, C3, and PGAM5 in human ICH brain tissues. Scale bar: 50 µm. G,H) The analysis and quantification of PGAM5 protein were carried out in primary astrocytes treated with CHX, with or without ITC stimulation (*n* = 3 per group, two‐way ANOVA). The asterisk at the top indicates the comparison between the two groups at identical time points. I) Detection of PGAM5 ubiquitination levels in primary astrocytes treated with MG132, with or without ITC stimulation (*n* = 3 per group). J) Schematic diagram of immunoprecipitation coupled with mass spectrometry (IP‐MS). (K) HEK293T cells were co‐transfected with Myc‐tagged USP11 or empty vector alongside Flag‐tagged PGAM5. Cell lysates underwent immunoprecipitation with anti‐Flag or anti‐Myc antibodies, followed by immunoblotting using antibodies for detection. L) The protein level of PGAM5 in HEK293T cells with or without USP11 overexpression (*n* = 6 per group, Student's *t*‐test). M) Western blot analysis and quantification of PGAM5 in *Usp11*‐silenced primary astrocytes pretreated with MG132 or Baf with or without ITC stimulation (*n* = 3 per group, one‐way ANOVA). N) HEK293T cells expressing Flag‐PGAM5 and Myc‐USP11 were co‐transfected with HA‐tagged ubiquitin (Ub), K48R‐only, or K63R‐only mutant plasmids as indicated. Data are presented as means ± SD. *p < 0.05, **p < 0.01, ***p < 0.001.

To explore potential mechanisms regulating neurotoxic astrocyte reactivity, astrocytes were isolated from mice on day 3 post‐ICH by flow cytometry for proteomic sequencing (Figure S1A, Supporting Information). Subsequently, we identified 646 differentially expressed genes (DEGs) following ICH (**Figure** **1**A), primarily enriched in immune‐inflammatory responses, cell death, and immune cell migration processes (Figure S1B). Notably, mitochondria play crucial roles in regulating oxidative stress, inflammation, and cell death, and their dysfunction underlies the pathogenesis of diverse disorders including neurodegenerative, metabolic, cardiovascular, and autoimmune diseases.^[^ ^24^, ^25^ ^]^ Therefore, we specifically analyzed mitochondrial gene expression changes among all DEGs post‐ICH, identifying PGAM5 as the most significantly altered mitochondrial gene compared to the control group (Figure 1B).

To further validate PGAM5 expression changes in neurotoxic astrocytes following ICH, we initially assessed its protein levels in astrocytes isolated from whole brain tissues of ICH mice exhibited significant elevation in the brain at 24 h post‐ICH, with a peak at 72 h and sustained elevation for at least 16days (Figure 1C). Moreover, immunostaining of mouse brains on day 3 post‐ICH also confirmed that ICH specifically upregulates PGAM5 in neurotoxic astrocytes (Figure 1D). Furthermore, to obtain more compelling evidence, we assessed the expression levels of PGAM5 in astrocytes isolated from surgically resected brain contusion tissues from ICH patients alongside control samples obtained during deep‐seated tumor resections in non‐ICH individuals (Figure S1C, Supporting Information). In line with observations in mice, Western blot analysis demonstrated marked elevation of PGAM5 levels in astrocytes isolated from ICH‐affected brain tissues relative to those in control samples (Figure 1E). Meanwhile, immunofluorescence co‐labeling of PGAM5 and C3 with GFAP revealed upregulated PGAM5 expression in neurotoxic astrocytes post‐ICH and demonstrated its concurrent association with these cells (Figure 1F).

Despite significant PGAM5 protein upregulation, interestingly, unchanged mRNA levels were detected in astrocytes following ICH in patients and in mice (Figure S1D,E, Supporting Information). Primary astrocytes were cultured and stimulated with IL‐1α, TNF‐α, and C1q (ITC) to induce a neurotoxic astrocyte phenotype, as previously described.^[^ ^7^, ^26^ ^]^ Then, we evaluated the mRNA expression of genes that mark neurotoxic astrocytes (*C3*, *Serping1*, *Psmb8*) after ITC stimulation and observed a significant increase compared to the control group (Figure S1F, Supporting Information). In contrast, the expression of neuroprotective astrocyte marker genes (*S100a10*, *Ptx3*, *Tgm1*) remained unaffected, indicating the successful establishment of an in vitro model for the reactivity of neurotoxic astrocytes (Figure S1G, Supporting Information). Consistent with in vivo observations, cultured primary astrocytes treated with ITC stimulation similarly exhibited augmented PGAM5 expression without significant alterations in its mRNA levels (Figure S1H,I, Supporting Information). This finding eliminates altered transcriptional regulation as a primary reason for the detected increase in protein abundance. Collectively, PGAM5 protein elevation occurs in neurotoxic astrocytes post‐ICH independently of transcriptional upregulation.

### 2.2 USP11 Deubiquitinates PGAM5 to Suppress its Ubiquitin‐Proteasome Degradation

To better understand the factors contributing to the difference between mRNA and protein levels of PGAM5, primary astrocyte cells were treated with cycloheximide (CHX, a protein synthesis inhibitor) under both vehicle and ITC stimulation. The findings indicated that ITC stimulation significantly extended the half‐life of PGAM5 protein (Figure 1G,H), implying that the variations in PGAM5 expression during astrocyte reactivity are a result of modified protein stability. Protein degradation primarily occurs through two mechanisms: the lysosomal‐autophagy route and the proteasomal‐ubiquitin route. Notably, upon CHX treatment, MG132 (a proteasome inhibitor) elevated PGAM5 levels in primary astrocytes, whereas Baf (a lysosomal inhibitor) produced no such effect (Figure S1J,K, Supporting Information). Concurrently, ITC and MG132 stimulations substantially decreased PGAM5 ubiquitination (Figure 1I). Consequently, decreased proteasomal degradation of PGAM5 leads to increased expression of PGAM5 in neurotoxic astrocytes.

To identify ubiquitination or deubiquitination enzymes regulating PGAM5 expression in this process, immunoprecipitation coupled with mass spectrometry (IP‐MS) was employed to identify PGAM5‐interacting proteins, revealing USP11 as a potential interacting partner (Figure 1J). USP11 is a deubiquitinase with high expression levels in the human brain.^[^ ^27^ ^]^ Supplementary co‐immunoprecipitation (Co‐IP) experiments performed in HEK293T cells with epitope‐tagged constructs validated these interactions. Flag‐tagged PGAM5 demonstrated robust co‐precipitation with Myc‐tagged USP11 (Figure 1K). USP11 overexpression markedly boosted PGAM5 protein levels (Figure 1L), with no impact on its mRNA expression (Figure S1L, Supporting Information). We then performed systematic protein stability assays in *Usp11*‐silenced (si*Usp11*) primary astrocytes cells with the proteasome inhibitor MG132 and the lysosome inhibitor Baf following ITC challenge. We then found that si*Usp11* significantly reduced PGAM5 expression, an effect reversed by proteasome inhibitor MG132. In contrast, lysosomal protease inhibitor Baf treatment showed no such effect (Figure 1M). Additionally, we mutated ubiquitin lysine residues to evaluate USP11's impact on PGAM5 polyubiquitination. The K48R ubiquitin mutant completely inhibited USP11‐mediated PGAM5 polyubiquitination, demonstrating that USP11 stabilizes PGAM5 by specifically cleaving K48‐linked polyubiquitin chains (Figure 1N). Furthermore, the transcriptomic and protein levels of USP11 were evaluated in both the ICH mouse model and ITC‐stimulated astrocytes. No significant alterations in USP11 expression were observed under these conditions (Figure S1M–Q, Supporting Information). However, Co‐IP experiments showed an enhanced interaction between USP11 and PGAM5 (Figure 1K), indicating that the enhanced USP11‐PGAM5 interaction is the primary mechanism responsible for the effect of USP11. Collectively, USP11 specifically elevates PGAM5 expression in neurotoxic astrocyte phenotype after ICH via deubiquitination‐mediated protein stabilization.

### 2.3 Astrocyte‐Specific Knockout of Pgam5 or Usp11 Promotes Neurological Recovery after ICH

**Figure 2:**
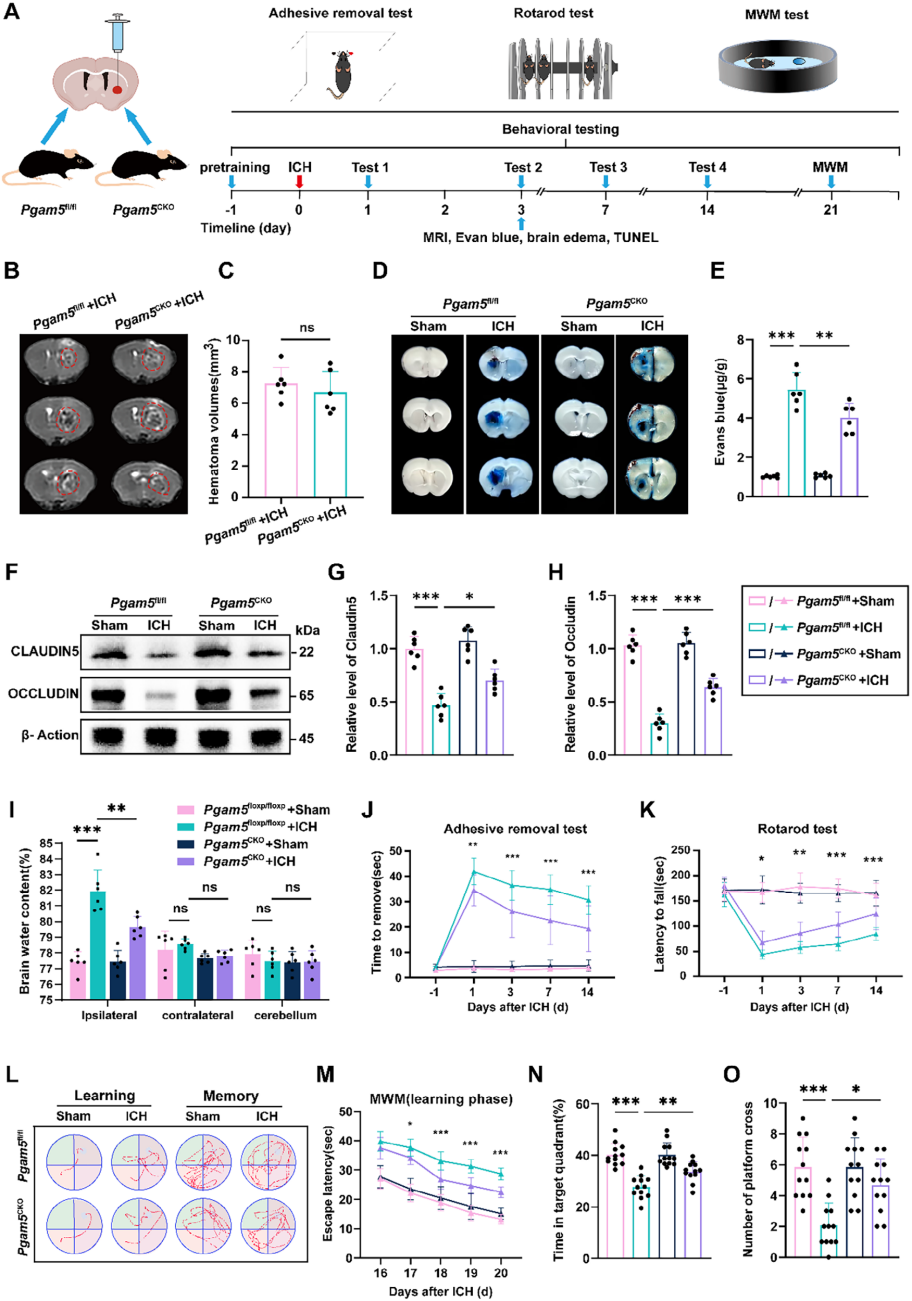
*Pgam5* ablation in astrocytes enhances neurological function following ICH. (A) Flow chart for neurological function assessment. B,C) Sample MRI scans and measurement of brain lesion size 3 days post‐ICH (*n* = 6 per group, Student's *t* test). D,E) Illustrative brain sections with Evan blue and their quantification from *Pgam5* ^flox/flox^ and *Pgam5* ^CKO^ mice at day 3 after ICH (*n* = 6 per group, Student's *t* test). F–H) Western blot analysis and quantification of the expression of CLAUDIN‐5 and OCCLUDIN in each group at day 3 after ICH (*n* =  6 per group, one‐way ANOVA). I) The quantification of brain water content at 3d after ICH (*n* = 6 per group, one‐way ANOVA). (J‐K) Adhesive removal J) and rotarod test K) were used to assess sensorimotor functions in *Pgam5* ^flox/flox^ and *Pgam5* ^CKO^ ICH mice (*n* = 12 per group, two‐way ANOVA). L–O) Learning and memory capabilities were evaluated using the MWM test (*n* = 12 per group, M for two‐way ANOVA, N for one‐way ANOVA, O for Kruskal–Wallis H test). Data are presented as means ± SD. *p < 0.05, **p < 0.01, ***p < 0.001. Behavioral analyses: Asterisks indicate daily comparisons between the two groups (ICH‐*Pgam5* ^flox/flox^ group versus ICH‐*Pgam5* ^CKO^ group).

To elucidate the role of PGAM5 modulation in astrocytes on brain damage post‐ICH, we generated astrocyte‐specific conditional *Pgam5*‐knockout (*Pgam5* ^CKO^) mice by crossing respective floxed alleles (*Pgam5* ^flox/flox^) with *Aldh1l1*‐Cre/ERT2 transgenic lines, followed by tamoxifen administration to induce gene‐specific deletions (Figure S1R, Supporting Information). Furthermore, we performed multiple experimental assessments on our established mouse model, including MRI, Evans Blue staining, brain water content measurement, behavioral tests, and the Morris water maze (MWM) test (**Figure** **2**A). MRI results showed similar hematoma volume at 24 h post‐ICH in *Pgam5* ^CKO^ mice, compared to that in *Pgam5* ^flox/flox^ mice (Figure 2B,C). Since blood‐brain barrier (BBB) impairment is essential in ICH‐related secondary brain injury, we assessed if *Pgam5* ^CKO^ mice could prevent BBB damage. The findings revealed a significant increase in Evans blue leakage in the ICH group, but *Pgam5* ^CKO^ mice greatly reduced this increase, suggesting that *Pgam5* ^CKO^ mice can safeguard BBB integrity from ICH damage (Figure 2D,E). Additionally, we discovered that the ICH group had lower levels of tight junction proteins (Claudin‐5 and Occludin) relative to the sham group, but this reduction was reversed in *Pgam5* ^CKO^ mice (Figure 2F–H). Meanwhile, brain water content analysis indicated that ICH‐induced brain oedema was significantly alleviated in *Pgam5* ^CKO^ mice compared to *Pgam5* ^flox/flox^ ICH mice (Figure 2I). Notably, *Pgam5* ^CKO^ ICH mice exhibited markedly reduced adhesive tape removal durations and prolonged rotarod latency relative to flox/flox ICH controls (Figure 2J,K). Subsequent MWM testing assessed whether therapeutic strategies against *Pgam5* alleviate cognitive deficits resulting from ICH (Figure 2L). During learning trials, *Pgam5* ^flox/flox^ ICH mice showed longer escape latencies relative to *Pgam5* ^CKO^ ICH mice (Figure 2M). In probe trials, *Pgam5* ^CKO^ mice with induced ICH exhibited greater occupancy of the target quadrant and more frequent platform crossings compared to *Pgam5* ^flox/flox^ ICH mice (Figure 2N,O).

Furthermore, we generated astrocyte‐specific *Usp11* knockout (*Usp11* ^CKO^) mice and found that astrocyte‐specific *Usp11* knockout similarly demonstrated significant reductions in brain water content (Figure S2A, Supporting Information), alleviation of sensorimotor deficits (Figure S2B,C, Supporting Information), and mitigation of cognitive impairments (Figure S2D–G, Supporting Information) post‐ICH. Consequently, targeted ablation of astrocyte‐specific *Pgam5* or *Usp11* exhibited the potential to facilitate neurological recuperation subsequent to ICH.

### 2.4 Targeted Knockout of Astrocyte‐Specific Pgam5 Attenuates Release of Proinflammatory Mediators and Neurotoxic Factors

**Figure 3:**
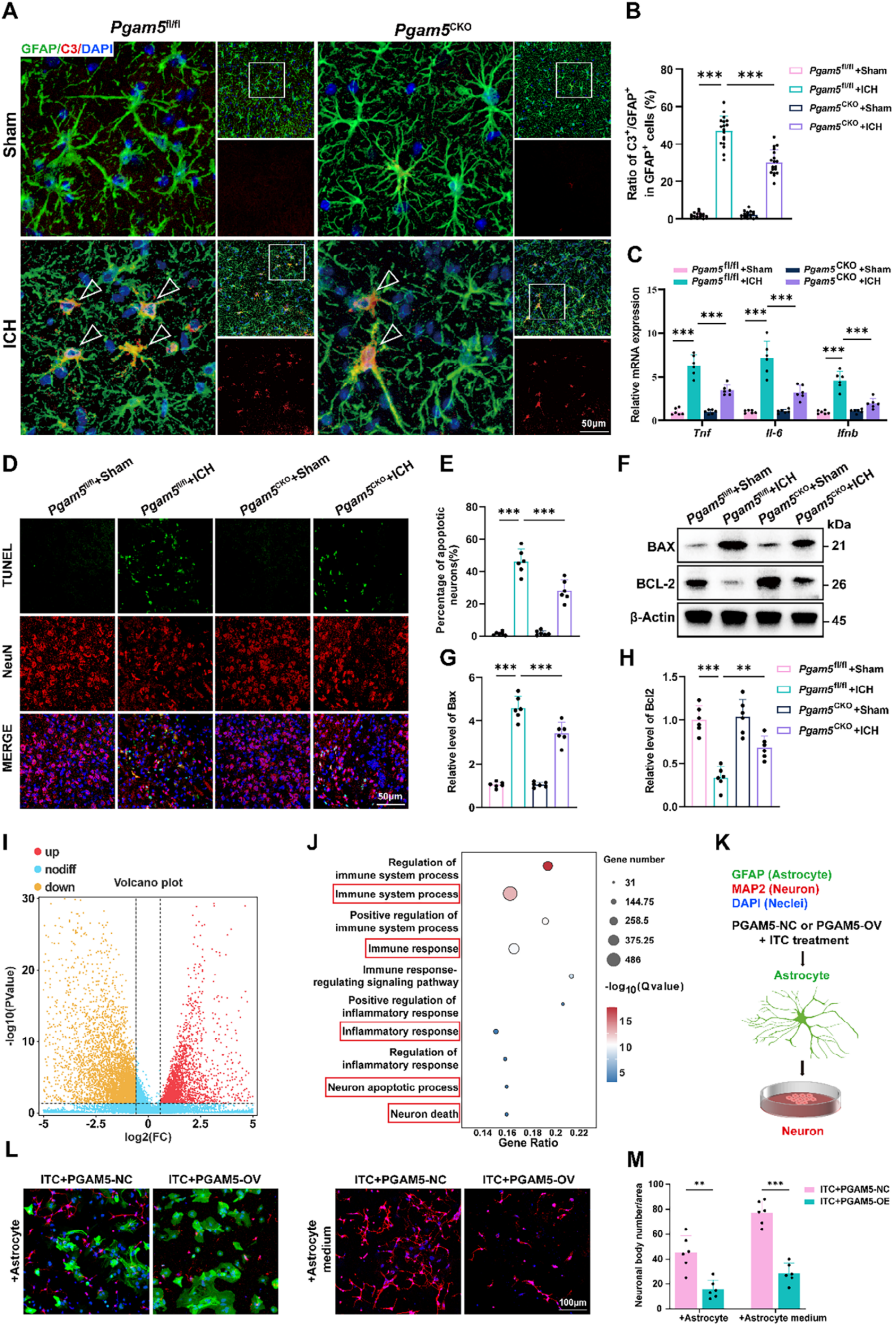
*Pgam5* ablation in astrocytes suppresses neurotoxic astrocytes reactivity and attenuates release of proinflammatory mediators and neurotoxic factors. A,B) Immunofluorescence labeling of GFAP and C3, along with statistical evaluation of C3 positive cells in GFAP^+^ cells at day 3 after ICH (*n* = 18 slices from 6 mice per group, one‐way ANOVA). Scale bar: 50 µm. C) PCR analysis of mRNA level of several inflammatory cytokines, including *Tnf*, *Il6* and *Ifnb* at day 3 after ICH (*n* = 6 per group, one‐way ANOVA). D) Representative TUNEL staining images in mice from each group. Scale bar: 50 µm. E) The measurement of TUNEL positive cells (*n* = 6 per group, one‐way ANOVA). F–H) Representative Western blot images and quantification of the expression of BAX and BCL‐2 in each group at day 3 after ICH (*n* =  6 per group, one‐way ANOVA). I) Differential gene distribution was revealed in the volcano plot. J) GO analysis following RNA‐seq. K) Schematic diagram of co‐culture between PGAM5‐NC and PGAM5‐OV primary astrocytes with primary neurons with ITC stimulation. L,M) Representative images and the quantification of the number of neuronal cell bodies following co‐culture with PGAM5‐NC or PGAM5‐OV primary astrocytes, or treatment with their conditioned medium (n = 6 per group, one‐way ANOVA). Scale bars:100 µm. Data are presented as means ± SD. *p < 0.05, **p < 0.01, ***p < 0.001.

Subsequently, immunofluorescence staining demonstrated that astrocyte‐specific knockout of *Pgam5* significantly inhibited neurotoxic astrocyte reactivity in the peri‐hematoma region of ICH mice (**Figure** **3**A,B). Quantitative analysis of inflammatory gene expression further demonstrated a noteworthy reduction in levels of ICH‐induced TNF, Il‐6, and IFNB in *Pgam5* ^CKO^ ICH mice versus *Pgam5* ^flox/flox^ ICH mice (Figure 3C). Given the known neurotoxicity associated with astrocytes, we subsequently explored how knocking out *Pgam5* specifically in astrocytes affects neuron survival after ICH. Encouragingly, TUNEL staining showed diminished neural apoptosis in *Pgam5* ^CKO^ mice (Figure 3D,E). At the same time, western blotting showed a clear increase in BAX and a decrease in BCL2 due to ICH, both of which were notably reversed in *Pgam5* ^CKO^ mice (Figure 3F–H).

Similarly, *Usp11* ^CKO^ ICH mice inhibited neurotoxic astrocyte reactivity (Figure S2H,I, Supporting Information), diminished release of inflammatory factors (Figure S2J, Supporting Information), and attenuated neuronal apoptosis (Figure S2K, Supporting Information) compared to the *Usp11* ^flox/flox^ ICH mice. However, the protective effect in *Usp11* ^CKO^ ICH mice was less pronounced than that in *Pgam5* ^CKO^ ICH mice. This difference may arise because USP11 regulates an additional downstream target that confers protection in ICH. Consequently, subsequent experiments investigating the mechanism focused on PGAM5.

Primary astrocytes were exposed to ITC and subsequently transfected with PGAM5‐overexpressing (PGAM5‐OV) or control adenovirus. Furthermore, transcriptomic profiles were then analyzed by RNA sequencing (RNA‐seq) to identify differentially expressed genes. Volcano plots were used to effectively display the distribution of DEGs (Figure 3I). Gene Ontology (GO) analysis revealed significant upregulation of inflammatory response and neurotoxicity‐related processes (Figure 3J).

Then we assessed the relative neurotoxic potential of PGAM5‐OV primary astrocytes with control primary astrocytes. In one approach, primary neurons were co‐cultured with either PGAM5‐OV or wild‐type primary astrocytes. Compared to co‐culture with wild‐type astrocytes, co‐culture with PGAM5‐OV primary astrocytes significantly decreased the number of surviving neuronal cells. In a second approach, exposure of primary neurons to conditioned medium harvested from PGAM5‐OV primary astrocytes also resulted in decreased neuronal cell survival (Figure 3K–M). Furthermore, after implementing two distinct co‐culture approaches, neurites exhibiting significant shortening were observed in PGAM5‐OV primary astrocytes compared to PGAM5‐NC primary astrocytes (Figure S3A, Supporting Information). Besides, following ITC treatment and identical co‐culture conditions, partial rescue of neuronal survival and morphology was observed upon si*Pgam5* transfection (Figure S3B–E, Supporting Information). Collectively, these results demonstrate that PGAM5 modulates astrocytic inflammatory responses and neurotoxicity.

### 2.5 Astrocytic Pgam5 Knockout Attenuated both Neutrophil Infiltration and Microglial Activation through the CCL5‐CCR5 Chemotactic Axis

**Figure 4:**
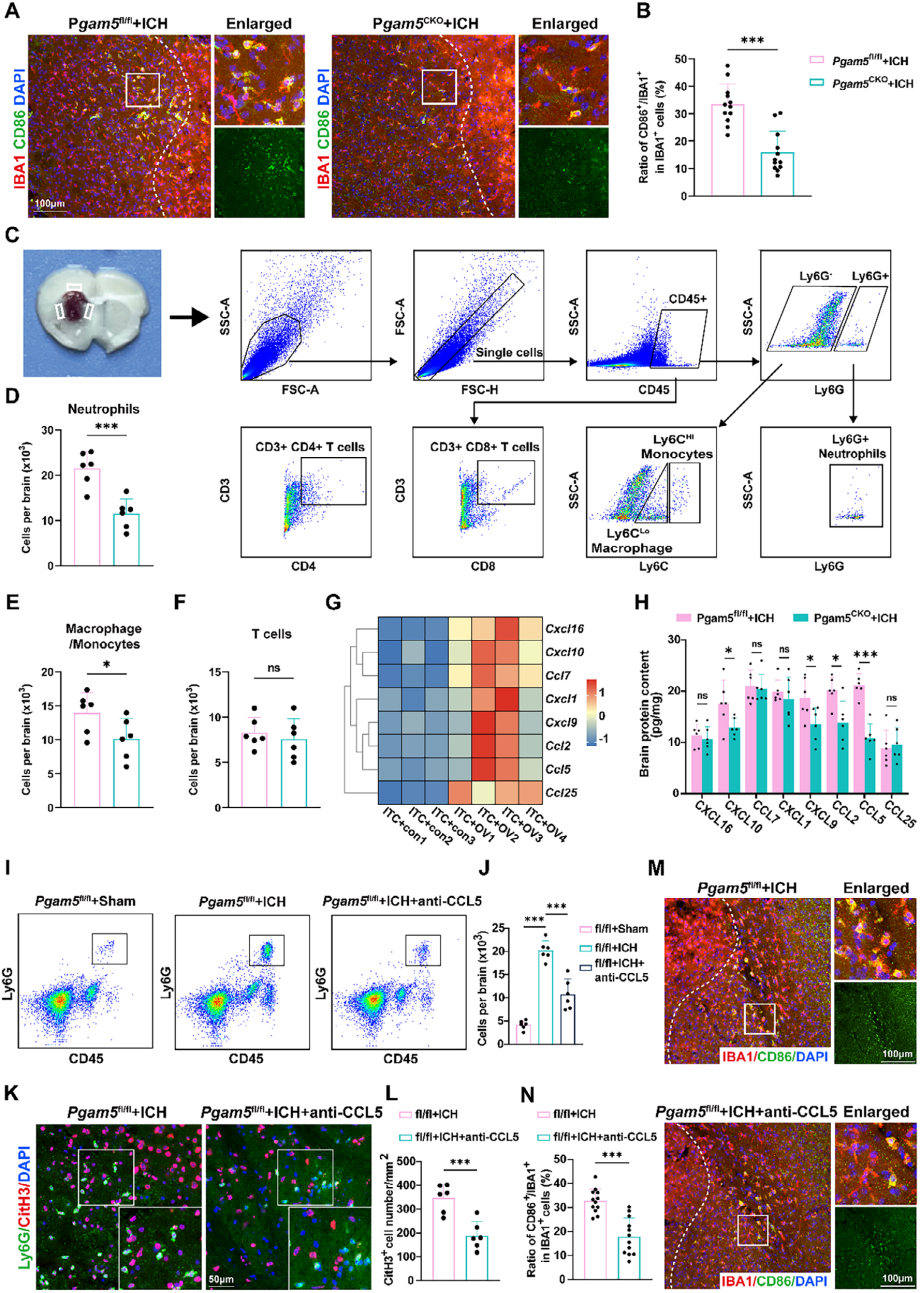
Astrocytic PGAM5 mediates neutrophil TEM and NET release via the CCL5‐CCR5 chemotactic axis. A,B) Immunofluorescence staining of IBA1 and CD86, and statistical analysis of CD86+ cells in IBA1+ cells after ICH (n = 12 slices from 6 mice per group, Student's t test). Scale bar: 100 µm. Hemorrhage borders are demarcated by distinct dashed lines. C) Example gating strategy for flow cytometric analysis of brain immune cells. D–F) Fluorescent‐conjugated surface antibodies were employed to distinguish distinct cell populations: D) Ly6G⁺ neutrophils, E) Ly6G^−^Ly6C⁺ macrophages/monocytes, and F) CD3e⁺ T cells (*n* = 6 per group, Student's *t* test). G) The heatmap demonstrated alterations in chemokine expression across experimental groups. H) Levels of chemokines CXCL16, CXCL10, CCL7, CXCL1, CXCL9, CCL2, CCL5, and CCL25 in the post‐ICH brain at 72 h were quantified via ELISA (*n* = 6 per group, Student's *t* test). I,J) Flow cytometry assessed the impact of anti‐CCL5 antibody on Ly6G⁺ neutrophil infiltration levels in mouse brains at 24 h after ICH (*n* = 6 per group, one‐way ANOVA). (K‐L) Quantification analysis of the CitH3^+^ cell number per mm^2^ (*n* = 6 per group, Student's *t*‐test). Scale bar: 50 µm. M,N) Representative micrographs of IBA1 and CD86 expression, along with quantitative analysis of CD86+ cells in IBA1+ cells following anti‐CCL5 treatment (*n* = 12 slices from 6 mice per group, Student's *t*‐test). Scale bar: 100 µm. Data are presented as means ± SD. *p < 0.05, **p < 0.01, ***p < 0.001.

Beyond exerting direct neurotoxicity, neurotoxic astrocytes remodel the brain's immune microenvironment by regulating microglial activity and peripheral immune cell infiltration, ultimately contributing to neuronal death through these indirect mechanisms.^[^ ^28^, ^29^ ^]^ Consequently, we investigated the impact of astrocyte‐specific *Pgam5* on the brain immune microenvironment. First, our further immunofluorescence staining demonstrated that astrocyte‐specific *Pgam5* knockout mitigated proinflammatory polarization of microglia (**Figure** **4**A,B). Subsequently, we examined by flow cytometry whether conditional *Pgam5* knockout alters peripheral immune cell brain infiltration. Primary gating discriminated CD45high and CD45int leukocyte populations (Figure 4C). Data demonstrated significantly reduced neutrophil proportions in *Pgam5* conditional knockout ICH mice versus *Pgam5* ^flox/flox^ ICH controls (Figure 4D). Moreover, the *Pgam5* ^CKO^ ICH mice showed a minor decrease in macrophage/monocyte cell counts (Figure 4E), and T cell subsets did not differ significantly from those in the *Pgam5* ^flox/flox^ ICH mice (Figure 4F). To determine whether significant disparities in neutrophil presence were attributed to elevated concentrations of systemic neutrophils in *Pgam5* ^CKO^ mice, peripheral blood specimens were analyzed to compare circulating levels. Similar neutrophil numbers were observed in the blood of *Pgam5* ^flox/flox^ and *Pgam5* ^CKO^ mice subjected to ICH (Figure S4A‐B, Supporting Information). These findings suggest that increased neutrophil accumulation within cerebral tissue of *Pgam5* ^CKO^ ICH mice, relative to *Pgam5* ^flox/flox^ ICH mice, stems not from heightened circulatory counts but from greater efficiency in neutrophil migration to the brain.

Recent studies have revealed that the CXCL and CCL families represent two major classes of chemokines whose core function involves orchestrating directional immune cell migration, playing crucial roles in immune surveillance, inflammatory responses, tissue homeostasis, and diverse pathological processes.^[^ ^30^, ^31^ ^]^ From our RNA‐seq data, we identified that Cxcl16, Cxcl10, Ccl7, Cxcl1, Cxcl9, Ccl2, Ccl5, and Ccl25 from these families were markedly upregulated upon PGAM5 overexpression and thus warrant further investigation (Figure 4G). We further validated these chemokines in a mouse ICH model. ELISA revealed that astrocyte‐specific knockout of *Pgam5* most prominently attenuated ICH‐induced upregulation of CCL5 expression in whole brain tissue, with less pronounced effects on other chemokines (Figure 4H). To determine the cellular origins of elevated CCL5, immunofluorescence co‐staining for CCL5 with cell‐specific markers (GFAP for astrocytes, IBA1 for microglia, and NeuN for neurons) was performed in the peri‐hematomal region of ICH mice. This data robustly confirms that CCL5 is primarily localized to GFAP positive astrocytes (Figure S4C,D, Supporting Information). These results demonstrate that PGAM5 specifically upregulates CCL5 expression in astrocytes following ICH. Interestingly, we observed high levels of *Ccl5* mRNA in microglia following ICH (Figure S4E,F, Supporting Information), suggesting a potential impediment in the translation process of *Ccl5* mRNA into protein, which warrants further investigation in future studies.

CCL5 is essential for regulating the inflammatory reactions of immune cells by binding to its receptor CCR5.^[^ ^32^, ^33^ ^]^ Subsequently, we found that a CCL5‐neutralizing antibody or a CCR5 antagonist significantly attenuated neutrophil infiltration (Figure 4I,J and Figure S4G,H, Supporting Information), and neutrophil extracellular traps (NETs) formation (Figure 4K,L and Figure S4I,J, Supporting Information). This is consistent with previous reports that astrocytes can recruit neutrophils and facilitate their infiltration via the CCL5‐CCR5 axis, thereby driving the pathogenesis of depression.^[^ ^34^ ^]^ We also found that either a CCL5‐neutralizing antibody or a CCR5 antagonist attenuated microglial proinflammatory polarization (Figure 4M,N and Figure S4K,L, Supporting Information). To further investigate the role of CCL5 and CCR5 in microglial activation, conditioned medium (CM) was collected from primary astrocytes following ITC stimulation. High levels of CCL5 were detected in the CM (Figure S4M, Supporting Information). Exposure of primary microglia to this CM resulted in increased expression of proinflammatory factors, including *Tnf‐α*, *Il‐6* and *Ifnb*. Notably, co‐treatment of primary microglia with CM and CCL5‐neutralizing antibody, or with CM and CCR5 antagonist, significantly attenuated microglial activation (Figure S4N, Supporting Information). Together with the in vivo findings, these results provide further evidence that astrocyte‐secreted CCL5 promotes microglial activation primarily through the CCR5‐mediated signaling pathway. Collectively, deletion of *Pgam5* in astrocytes suppressed neutrophil recruitment and microglial activation, mediated through the CCL5‐CCR5 chemotactic axis.

### 2.6 PGAM5 Promoted mtDNA Leakage and Activated the cGAS‐STING Pathway after ICH

**Figure 5:**
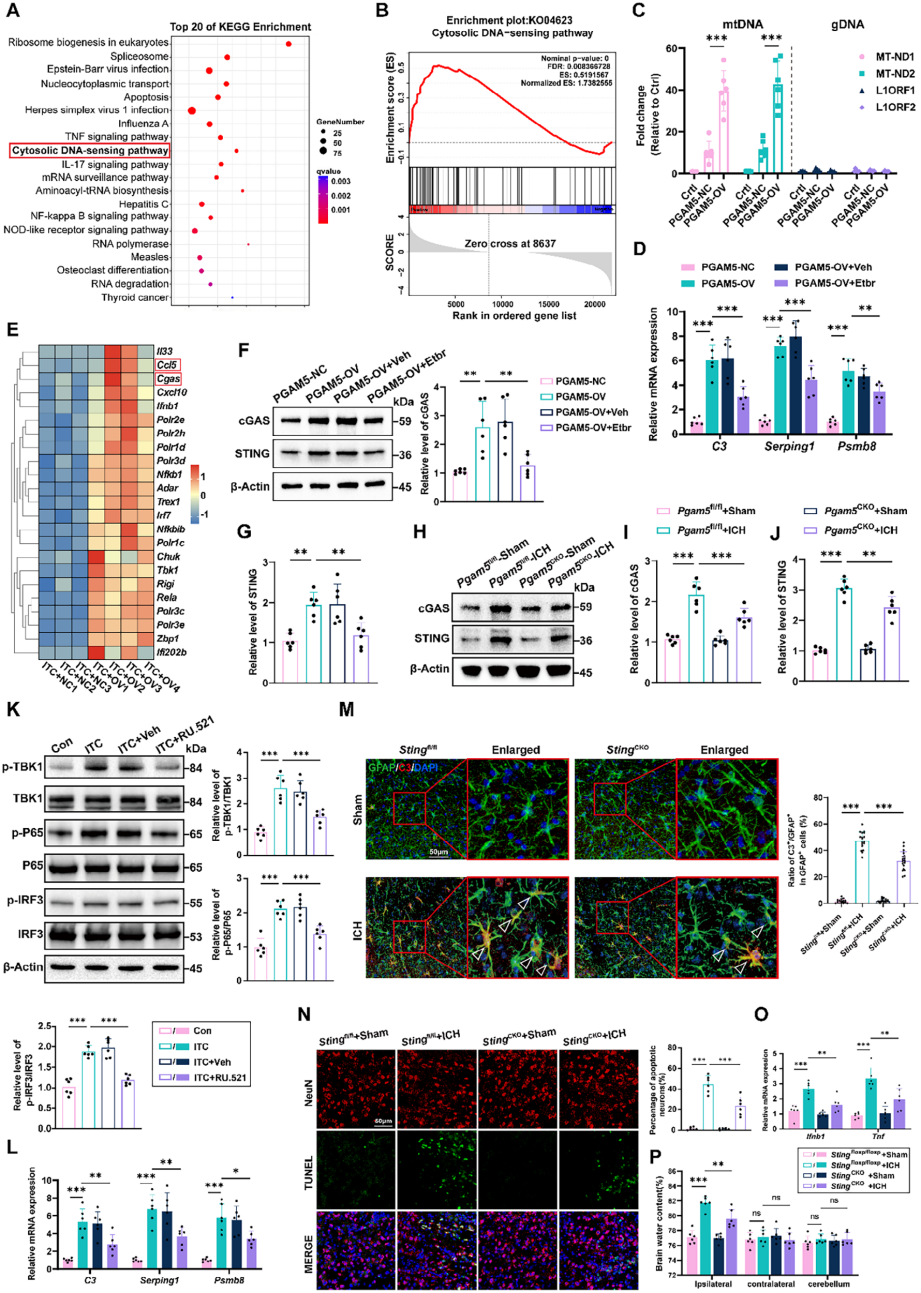
PGAM5 drives mtDNA liberation and subsequent cGAS‐STING pathway activation following ICH. A) KEGG analysis following RNA‐seq. B) GSEA of cytosolic DNA‐sensing pathway based on KEGG. C) qPCR validation confirmed mtDNA transcripts (MT‐ND1/MT‐ND2) without detecting nuclear‐derived LINE1 elements (L1ORF1, L1ORF2) (*n* =  6 per group, one‐way ANOVA). D) qPCR analysis of mRNA levels of astrocyte A1 markers (*C3, Serping1, Psmb8*) in PGAM5‐OV primary astrocytes with or without Etbr treatment (*n* = 6 per group, one‐way ANOVA). E) The heatmap demonstrated differential gene expression in the cytosolic DNA‐sensing pathway. F–G) Western blot analysis of the expression level of cGAS‐STING in ITC and PGAM5‐OV treated astrocytes with or without treated with Etbr (*n* =  6 per group, one‐way ANOVA). H–J) Western blot analysis and quantification of the expression of cGAS and STING in each group mice under diverse treatment regimens (*n* =  6 per group, one‐way ANOVA). K) Western blot analysis of the expression level of cGAS‐STING pathway in ITC treated astrocytes with or without RU.521 treatment (*n* =  6 per group, one‐way ANOVA). L) PCR analysis of mRNA level of neurotoxic astrocyte marker including *C3*, *Serping1*, *Psmb8* in ITC treated astrocytes with or without treated with RU.521 (*n* =  6 per group, one‐way ANOVA). M) Dual‐immunofluorescence co‐localization analysis of GFAP and C3, with quantification of C3⁺/GFAP⁺ cell ratios, was performed in *Sting* ^fl/fl^ versus *Sting* ^CKO^ mice post‐ICH or sham surgery (*n* = 18 slices from 6 mice per group, one‐way ANOVA). Scale bar: 50 µm. N) TUNEL‐positive cells in mouse brain sections were quantitatively evaluated at 72 h post‐ICH. Scale bar: 50 µm. Results reflect neuronal apoptosis metrics based on TUNEL⁺/NeuN⁺ cell enumeration (n = 6 per group, one‐way ANOVA). O) PCR analysis of mRNA level of several inflammatory cytokines including *Ifnb1, Tnf*. P) Analysis of brain water content 72 h post‐ICH in STING^fl/fl^ and STING^CKO^ mice (*n* = 6 per group, one‐way ANOVA). Data are presented as means ± SD. *p < 0.05, **p < 0.01, ***p < 0.001.

Notably, based on Kyoto Encyclopedia of Genes and Genomes (KEGG) and Gene Set Enrichment Analysis (GSEA) of RNA‐seq data, we found that PGAM5 significantly activated the cytosolic DNA‐sensing pathway (**Figure** **5**A,B). While DNA normally resides in the nucleus and mitochondria, aberrant release of mtDNA into the cytosol potently induces immune‐inflammatory responses.^[^ ^35^ ^]^ To ascertain the source of cytosolic DNA in response to PGAM5, primary astrocytes were treated with ITC and subsequently transfected with either PGAM5‐OV adenovirus or control adenovirus. This demonstrated that PGAM5‐OV increased mtDNA abundance without evidence of abundant nuclear DNA sequences such as LINE1 elements (Figure 5C). Then, primary astrocytes were rendered mtDNA‐deficient through 3‐week treatment with ethidium bromide (EtBr), a mtDNA depletion agent. Mitochondrial gene expression analysis by qPCR confirmed successful mtDNA depletion (Figure S5A, Supporting Information). Following mtDNA depletion, we observed attenuated elevation of neurotoxic astrocyte marker genes induced by PGAM5‐OV (Figure 5D). Collectively, these results demonstrate that PGAM5 exacerbates neurotoxic astrocyte reactivity by promoting mtDNA leakage.

mtDNA is detected by specific DNA‐sensing pathway receptors such as cGAS, AIM2, and TLR9, which trigger cytokine release and may induce pyroptosis, a lytic inflammatory cell death pathway.^[^ ^36^ ^]^ Subsequently, we performed further analysis of DEGs within the DNA‐sensing pathway in RNA‐seq data, revealing that *Pgam5* significantly modulated cGAS expression (Figure 5E). mtDNA activates cGAS, a cytosolic sensor whose activation initiates STING‐dependent immunity, thus establishing the cGAS‐STING axis as a critical regulator in autoimmunity and inflammation.^[^ ^37^ ^]^ mtDNA depletion attenuated cGAS‐STING pathway activation demonstrating that PGAM5 mediates proinflammatory effects through mtDNA‐dependent cGAS‐STING pathway (Figure 5F–G). In vivo, we observed consistent results in *Pgam5* ^flox/flox^ and *Pgam5* ^CKO^ ICH mice (Figure 5H–J). Further experimental validation also revealed an upregulation of cGAS and STING protein levels in primary astrocytes following ITC stimulation alone (Figure S5B–D). Notably, STING recruits TBK1, leading to the phosphorylation of IRF3 and NF‐κB. which subsequently translocate to the nucleus and induce transcription of proinflammatory genes. In line with the elevated expression of cGAS‐STING, we observed a significant upregulation of phosphorylated TBK1, NF‐κB, and IRF3 levels following ITC (Figure S5E–G, Supporting Information).

We then proceeded to investigate whether the activation of the cGAS‐STING pathway is directly related to the reactivity of neurotoxic astrocytes. Interestingly, following ITC treatment of primary astrocytes, subsequent treatment with the cGAS inhibitor RU.521 effectively prevented phosphorylation of TBK1, NF‐κB, and IRF3, decreased mRNA levels of neurotoxic astrocyte marker genes (Figure 5K,L), attenuated neuronal loss and reduced inflammatory cytokine production (Figure S5H–J, Supporting Information).

In vivo, we generated *Sting* ^CKO^ mice and observed that *Sting* depletion alleviated both neurotoxic astrocyte reactivity and neuronal loss after ICH (Figure 5M,N). Similarly, this intervention markedly decreased the production of inflammatory cytokines and brain edema in ICH mice (Figure 5O,P). These findings provide compelling evidence for the crucial role of cGAS‐STING pathway in mediating the reactivity of neurotoxic astrocytes.

### 2.7 Elevated PGAM5 Promotes mPTP Opening and Enables Drp1 Dephosphorylation thus Amplifying mtDNA Leakage

**Figure 6:**
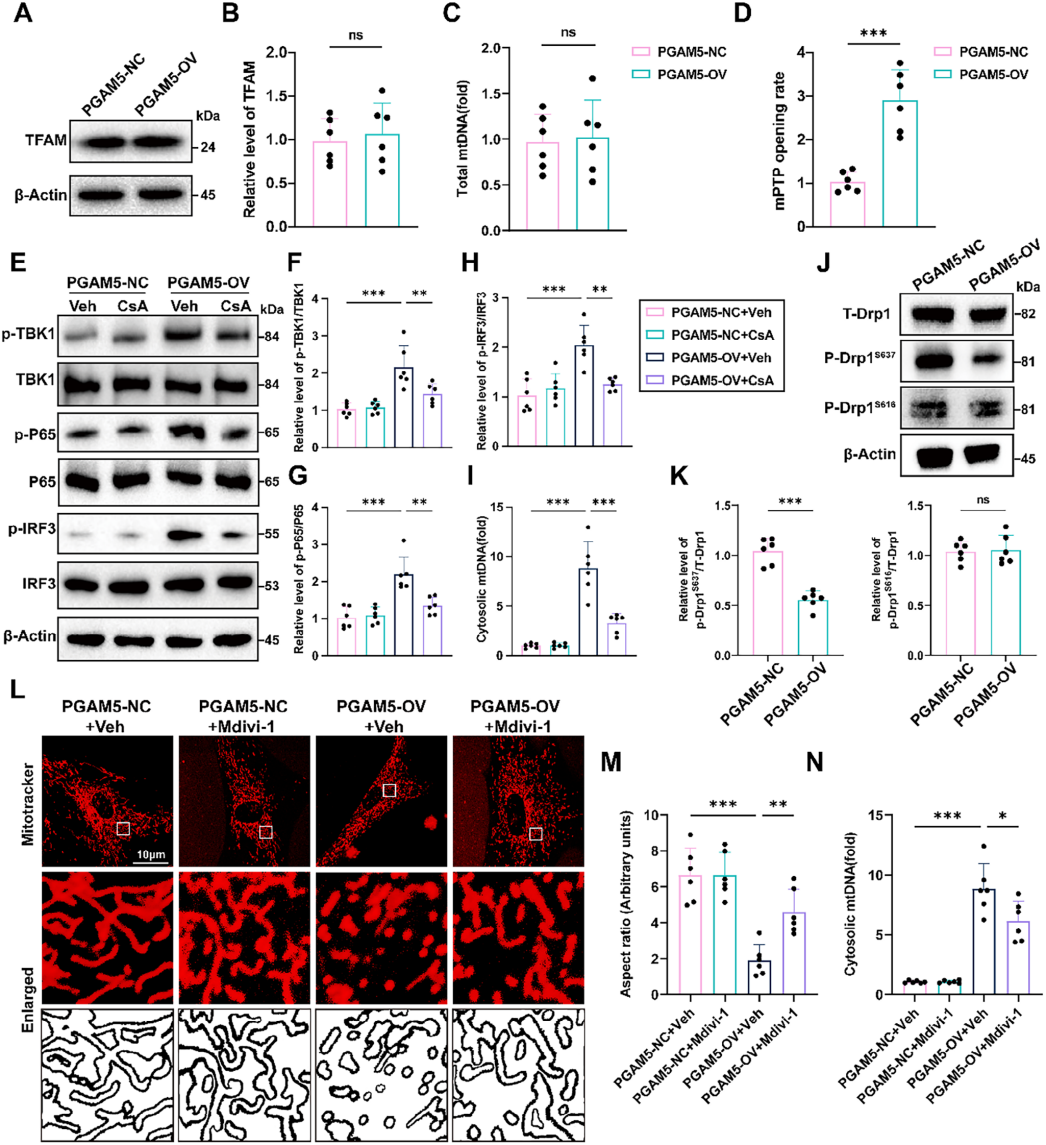
PGAM5 promotes mtDNA leakage in neurotoxic astrocytes through the opening of mPTP. A,B) Western blot analysis of TFAM expression (*n* =  6 per group, Student's *t*‐test). C) Quantification of total mtDNA (*n* =  6 per group, Student's *t*‐test). D) Quantification of mPTP opening rate in cultured PGAM5‐OV primary astrocytes (*n* = 6 per group, Student's *t*‐test). E–H) The effects of pharmacological in activation of the mPTP using CsA on PGAM5 induced activation of cGAS‐STING pathway (*n* =  6 per group, one‐way ANOVA). I) Cytosolic mtDNA content was quantitated via qPCR in each group (*n* =  6 per group, one‐way ANOVA). J,K) Western blot assay of the effects of PGAM5‐OV on the expression of Drp1, p‐Drp1^S637^ and p‐Drp1^S616^ and the related statistical analysis (*n* = 6 per group, Student's *t*‐test). L,M) Mitochondrial morphometric parameters were analyzed in primary astrocytes treated with PGAM5‐OV and Mdivi‐1. Fragmentation indices were quantified via aspect ratio measurements following MitoTracker staining (*n* =  6 per group, one‐way ANOVA). Scale bar: 10 µm. N) Cytosolic mtDNA levels were assessed using qPCR in each group (n =  6 per group, one‐way ANOVA). Data are presented as means ± SD. *p < 0.05, **p < 0.01, ***p < 0.001.

Then, we sought to investigate the underlying mechanisms through which PGAM5 induces the release of mtDNA. Transcription factor A, mitochondrial (TFAM), known to be the most abundant protein associated with mtDNA, plays a pivotal role in maintaining mtDNA structure, transcription, and replication. Disruption of TFAM function leads to disturbances in mtDNA biosynthesis and stability, consequently triggering mtDNA leakage. Surprisingly, our analysis revealed that TFAM levels remained unaltered (**Figure** **6**A,B). Simultaneously, we observed that total mtDNA copy numbers were not elevated in PGAM5‐OV astrocytes (Figure 6C). This prompted us to explore alternative mechanisms, specifically focusing on the destabilization and opening of the mPTP, which has been implicated in regulating mtDNA leakage. Intriguingly, our findings demonstrated a significant induction of mPTP opening upon PGAM5‐OV (Figure 6D). To further validate the role of mPTP in PGAM5‐mediated mtDNA leakage, we employed cyclosporin A (CsA), a pharmacological agent known to inhibit mPTP activity. Strikingly, treatment with CsA effectively abolished PGAM5‐induced mtDNA leakage into the cytoplasm, consequently attenuating the activation of the cGAS‐STING pathway (Figure 6E–I) and the subsequent expression of inflammatory cytokines (Figure S6A, Supporting Information). We further investigated the potential link between PGAM5 and Drp1. Intriguingly, we found that PGAM5 specifically modulated the dephosphorylation of Drp1 at Ser637, but not its phosphorylation at Ser616 (Figure 6J,K). Furthermore, PGAM5‐mediated Drp1 dephosphorylation induced mitochondrial fragmentation (Figure 6L,M) and mtDNA leakage (Figure 6N) in primary astrocytes, both of which were partially reversed by Mdivi‐1 treatment (a Drp1 inhibitor). Moreover, the relationship between Drp1 dephosphorylation and mPTP opening was investigated. PGAM5‐OV in primary astrocytes induced significant mPTP opening. Concomitant treatment of PGAM5‐OV astrocytes with Mdivi‐1, an inhibitor of Drp1, partially attenuated the mPTP opening (Figure S6B, Supporting Information). In contrast, application of the mPTP inhibitor CsA alongside PGAM5‐OV resulted in no significant change in Drp1 dephosphorylation (Figure S6C–E, Supporting Information). Furthermore, we found that co‐administration of CSA and Mdivi‐1 more effectively attenuated mtDNA leakage induced by PGAM5‐OV following ITC stimulation (Figure S6F, Supporting Information). These results suggest that the two processes operate primarily in parallel, both directly contributing to mtDNA leakage, but also exhibit unidirectional crosstalk. Collectively, these results provide compelling evidence that PGAM5 promotes concurrent mPTP opening and Drp1 dephosphorylation, synergistically enhancing mtDNA leakage.

### 2.8 Engineered EVs Effectively Deliver P*gam5* siRNA to Pathological Brain Tissue and Promotes Neurological Recovery after ICH

**Figure 7:**
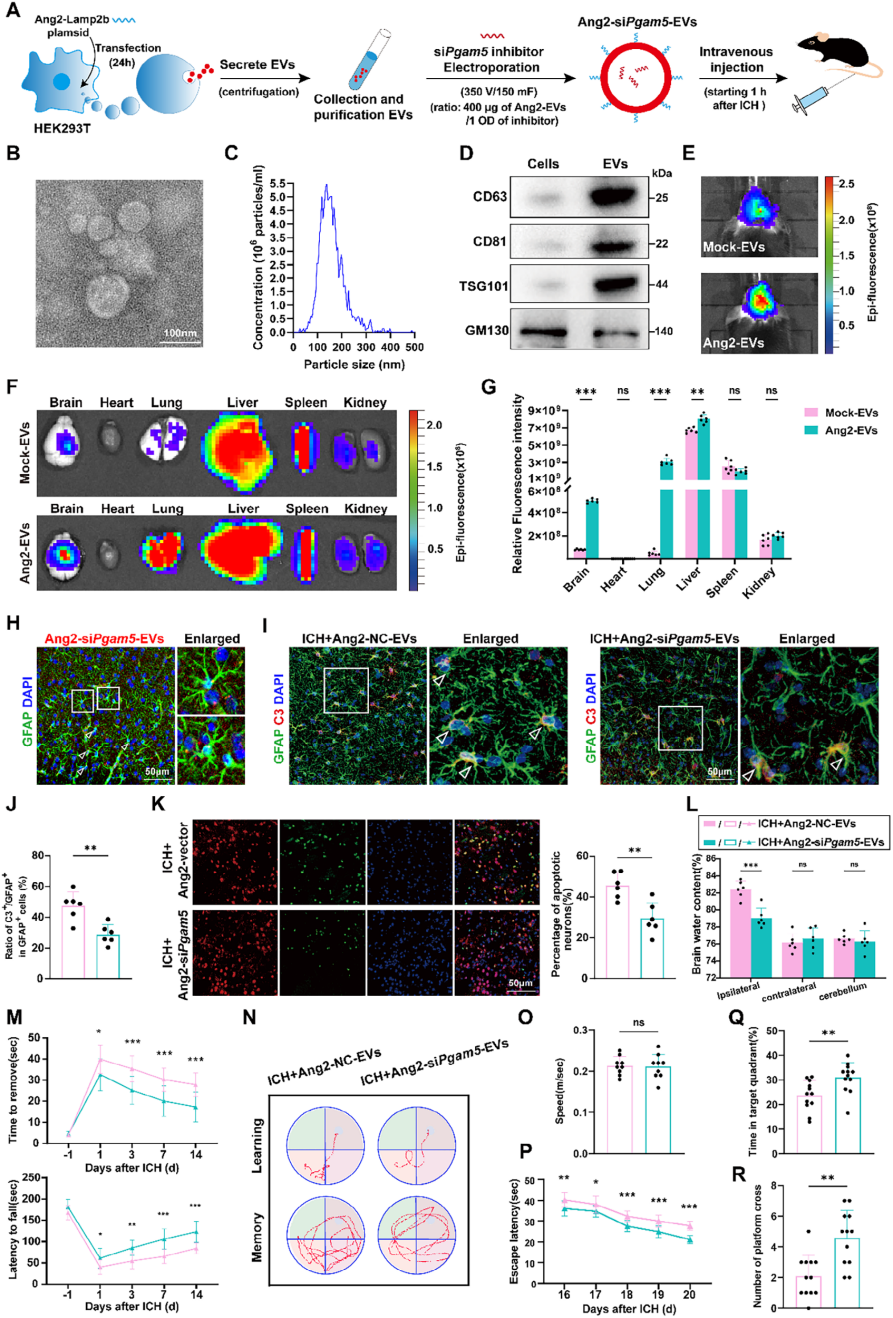
Engineered EVs deliver *Pgam5* siRNA precisely to astrocytes facilitating neurological functional recovery after ICH. A) Schematic overview of the manufacturing workflow for engineered EVs enabling therapeutic si*Pgam5* delivery. B) Representative transmission electron micrograph (TEM) depicting the ultrastructural morphology of isolated extracellular vesicles. Scale bar: 100 nm. C) Histograms illustrating EV size distribution and particle concentrations (particles/mL) isolated from HEK293T cultures. D) WB analysis of the expression level of CD63, CD81, TSG101 and GM130 in HEK293T‐derived EVs. E,F) In vivo biodistribution of DiR‐labeled mock EVs or Ang2‐EVs within mice organ systems at 24 h post‐injection, assessed through fluorescence imaging. G) Quantitative assessment of fluorescence intensity was performed on mice organs (*n* = 6 per group, Student's *t*‐test). H) Confocal micrographs visualize DiR‐conjugated Ang2‐si*Pgam5*‐EVs, where red fluorescent spots denote EV distribution and green fluorescence identifies GFAP⁺ astrocytes. Scale bar: 50 µm. I,J) Dual‐immunofluorescence colocalization analysis of GFAP and C3 was conducted, followed by quantification of C3⁺/GFAP⁺ cellular indices post‐ICH (*n* = 6 per group, Student's *t*‐test). Scale bar: 50 µm. K) Representative images of TUNEL staining of the ICH+Ang2‐NC‐EVs group or ICH+Ang2‐si*Pgam5*‐EVs group (*n* = 6 per group, Student's *t*‐test). Scale bar: 50 µm. L) For the statistical analysis of brain edema (*n* = 6 per group, Student's *t*‐test). M) Neurological function scores in the ICH+Ang2‐NC‐EVs group or ICH+Ang2‐si*Pgam5*‐EVs group (*n* = 12 per group, two‐way ANOVA). N–R) Cognitive functions were quantified via the Morris Water Maze assessment (*n* = 12 per group, *P* for two‐way ANOVA, O and Q for Student's *t*‐test, *R* for Mann–Whitney test). Data are presented as means ± SD. *p < 0.05, **p < 0.01, ***p < 0.001. Behavioral analyses: Asterisks indicate daily comparisons between the two groups (ICH+Ang2‐NC‐EVs group versus ICH+Ang2‐si*Pgam5*‐EVs group).

Currently, there are no suitable drugs‐targeting *Pgam5*. Given this therapeutic gap, we considered siRNA‐based intervention. However, the inherent instability of siRNA necessitates advanced delivery strategies. Modified engineered EVs may act as effective drug delivery vehicles by virtue of their low immunogenicity, controllable biodegradability, negligible toxicity, and capability to penetrate the BBB.^[^ ^38^ ^]^ We therefore engineered EVs to encapsulate si*Pgam5* for brain delivery, evaluating their therapeutic potential. Furthermore, by modifying the EV surfaces with particular peptides, they can specifically target and transport materials to designated tissues and cells.^[^ ^39^ ^]^ Angiopep‐2 (Ang2) is an oligopeptide reported to penetrate the BBB and target astrocytes.^[^ ^40^, ^41^, ^42^ ^]^ Thus, we engineered astrocyte‐targeting Ang2‐EVs by conjugating the Ang2 to the Lamp2b membrane protein on HEK293T‐derived extracellular vesicles (**Figure** **7**A). The Ang2‐EVs were obtained from the cultured cell supernatant using gradient centrifugation and confirmed through electron microscopy, nanoparticle analysis, and western blot analysis (Figure 7B–D). To evaluate targeting efficiency of this drug delivery system, Ang2‐EVs and mock‐EVs were labeled with 200 µg DiR and administered intravenously to ICH mice. Fluorescence intensity and distribution were analyzed 24 h later using an IVIS imaging system. After 24 h of intravenous injection, the brain had a higher concentration of DiR‐labelled Ang2‐EVs compared to mock‐EVs, according to the results (Figure 7E–G). This suggested that Ang2‐EVs had better brain‐targeting capabilities than mock‐EVs. Next, si*Pgam5* was loaded into Ang2‐EVs using electroporation and administered to ICH mice via the tail vein. Immunofluorescence staining with DiR‐labeled Ang2‐si*Pgam5*‐EVs and Gfap demonstrated that Ang2‐si*Pgam5*‐EVs is efficiently taken up by astrocyte (Figure 7H). The engineered Ang2‐EVs exhibited excellent stability in vitro, with encapsulated siRNA remaining intact for over 24 h (Figure S7A, Supporting Information). Critically, in vivo administration of Ang2‐si*Pgam5*‐EVs led to a significant knockdown of PGAM5 protein levels in the brain (Figure S7B, Supporting Information), demonstrating the functional stability and efficacy of the delivery system.

Next, we explored if Ang2‐si*Pgam5*‐EVs could reduce brain injury caused by ICH and enhance functional recovery in mice. Three days after ICH, neurotoxic astrocyte reactivity was significantly reduced after Ang2‐si*Pgam5*‐EVs treatment compared to Ang2‐NC‐EVs treatment (Figure 7I,J). This therapeutic intervention markedly decreased the TUNEL‐positive cell ratio and brain edema in ICH mice (Figure 7K,L). In line with that, behavioral assessments and the MWM test demonstrated that Ang2‐si*Pgam5*‐EVs treatment significantly improved sensorimotor function (Figure 7M) and enhanced memory (Figure 7N–R) in ICH‐affected mice. Collectively, these results suggest that Ang2‐si*Pgam5*‐EVs treatment aids in recovery after ICH.

## 3 Discussion

Through integrated clinical sample analysis and systematic validation using in vivo and in vitro models, this study establishes five novel findings: 1) PGAM5 serves as a key regulator driving neurotoxic astrocyte reactivity following ICH; 2) USP11 is identified as a novel deubiquitinase of PGAM5, mediating elevated PGAM5 expression in neurotoxic astrocytes; 3) PGAM5 activity in neurotoxic astrocytes not only promotes their own neurotoxicity but also modulates neutrophil infiltration and microglial activation via the CCL5‐CCR5 chemotactic axis; 4) PGAM5 promotes neurotoxic astrocyte reactivity by facilitating mtDNA leakage (through promoting mPTP opening and Drp1‐mediated mitochondrial fission) and thus activating the cGAS‐STING pathway; and 5) Extracellular vesicle‐mediated delivery of *Pgam5* siRNA significantly inhibited neurotoxic astrocyte reactivity and ameliorated neurological function in vivo.

Astrogliosis is a crucial pathological characteristic of ICH, involving molecular, morphological, and functional alterations in astrocytes during both acute and progressive pathological states. Astrocyte reactivity's influence on disease outcomes is contentious, as there is considerable evidence showing it can both obstruct and facilitate CNS recovery.^[^ ^28^, ^43^ ^]^ Reactive astrocytes exhibit diverse polarization states in different CNS diseases. These reactive astrocytes were later identified as “ neurotoxic astrocytes,” which have been found to be detrimental to synapses, indicating their potentially harmful functions.^[^ ^44^ ^]^ In contrast, neuroprotective astrocytes induced by ischemia upregulate numerous neurotrophic factors that promote neuronal survival and growth, which facilitate synaptic repair.^[^ ^45^ ^]^ Inhibition of the early polarization of astrocytes toward the neurotoxic astrocytes could be of great importance fosr patients' recovery. Numerous studies have established a link between mitochondrial dysfunction and the inflammatory response. In both our clinical ICH patients and mice ICH models, PGAM5, a distinctive protein phosphatase predominantly found in the mitochondrial membrane, was significantly upregulated in neurotoxic astrocytes during the acute phase of ICH and correlated with neurotoxic astrocyte reactivity. Meanwhile, we identified USP11 as a novel interaction protein of PGAM5, hindering the ubiquitination and degradation of PGAM5 in neurotoxic astrocytes. By utilizing mice with specific knockdown of *Pgam5* or *Usp11* in astrocytes, we further demonstrated that *Pgam5* or *Usp11* depletion can alleviate the reactivity of neurotoxic astrocytes and mitigate brain damage following ICH. Notably, the protective effect observed in astrocyte‐specific *Usp11* knockout mice was less pronounced than in *Pgam5* knockout mice. This attenuated efficacy suggests that USP11 likely regulates additional downstream targets beyond PGAM5, which may confer protective functions in the context of ICH. For instance, USP11 has been previously shown to deubiquitinate and stabilize proteins involved in both DNA repair and ferroptosis inhibition.^[^ ^46^, ^47^ ^]^ The net effect of *Usp11* knockout might therefore represent a balance between the beneficial inhibition of the PGAM5‐driven neurotoxic pathway and potential detrimental effects from stabilizing other unknown substrates. This intriguing possibility underscores the complexity of ubiquitin signaling, and highlights PGAM5 as a more specific and safer therapeutic target than USP11 for mitigating neurotoxic astrogliosis post‐ICH.

Neurotoxic astrocytes exhibit inherent neurotoxicity, releasing proinflammatory factors that exacerbate immune responses and secreting neurotoxic substances that directly damage neurons.^[^ ^48^ ^]^ Beyond these direct neurotoxic effects, these cells also reshape the brain's immune microenvironment by modulating microglial activity and facilitating peripheral immune cell infiltration, ultimately inducing neuronal death through these indirect mechanisms.^[^ ^49^, ^50^ ^]^ RNA‐seq revealed that PGAM5‐OV triggers the release of inflammatory factors and neurotoxic factors. Meanwhile, co‐culture experiments demonstrated that PGAM5 mediates astrocyte neurotoxicity toward neurons. Additionally, we found PGAM5 also regulates microglial activation and neutrophil infiltration. Activated microglia exacerbate neuronal death through multiple pathways, including proinflammatory cytokine release,^[^ ^51^ ^]^ synaptic stripping,^[^ ^52^ ^]^ and induction of excitotoxicity.^[^ ^53^ ^]^ Neutrophils further exacerbate neuronal death by releasing NETs and other cytotoxic factors.^[^ ^54^ ^]^ Previous studies demonstrated that astrocyte activation mediates the brain infiltration of neutrophils.^[^ ^34^ ^]^ In the present study, the same mechanism was observed in the ICH model. Astrocytic PGAM5 activates the cGAS–STING pathway, thus promoting the production and release of CCL5. The released CCL5 facilitates neutrophil infiltration by interacting with the CCR5 receptor on neutrophils. Therefore, astrocyte‐specific *Pgam5* deficiency not only directly attenuates astrocytic neurotoxicity but also indirectly reduces neuronal death by suppressing microglial activation and neutrophil infiltration. To elucidate how PGAM5 regulates immune remodeling, we analyzed RNA‐seq and identified the key chemokine CCL5 as a pivotal mediator in this process. CCL5‐neutralizing antibody or a CCR5 antagonist led to strong rescue of microglial activation induced by PGAM5, and reversed the promotion on microglial activation and neutrophil migration to a large extent. Taken together, the absence of *Pgam5* in astrocytes supports maintain neuronal health and creates a favorable setting for neuronal recovery, partly by modifying local inflammation and peripheral immune reactions in cases of ICH.

To clarify how PGAM5 enhances the reactivity of neurotoxic astrocytes, we analyzed RNA‐seq data and revealed significant activation of both the cytosolic DNA‐sensing and cGAS‐STING pathway. Cytosolic DNA, encompassing both microbial and endogenous sources such as extranuclear chromatin from genotoxic stress and mtDNA leakage, is recognized by specific receptors including cGAS, AIM2, and TLR9.^[^ ^55^ ^]^ The anomalous accumulation of mtDNA in the cytosol is detected by the cytosolic double‐stranded DNA (dsDNA) sensor, cGAS. This detection triggers cGAS to synthesize the second messenger 2′,3′‐cGAMP, which then binds to and activates STING, thereby initiating a downstream inflammatory signaling cascade.^[^ ^19^ ^]^ The cGAS‐STING pathway has primarily been identified as an important immune defense mechanism against microbial infections.^[^ ^56^ ^]^ Nonetheless, this pathway also plays a role beyond antimicrobial defense, as cGAS can sense and bind to dsDNA in a sequence‐independent manner, including self‐DNA derived from genomic or mtDNA.^[^ ^57^ ^]^ Recent studies have implicated the involvement of the cGAS‐STING pathway in both acute brain injury and chronic neurodegeneration diseases including ischemic brain injury, Huntington's disease and Parkinson's disease. For instance, the cGAS‐STING pathway in microglia promotes neuroinflammation and facilitates viral invasion in the CNS.^[^ ^58^, ^59^ ^]^ Similarly, another study revealed that pharmacological modulation of the cGAS‐STING pathway axis may effectively attenuate Alzheimer's disease progression.^[^ ^60^ ^]^ While the cGAS‐STING pathway has been extensively studied in microglia in the context of neuroinflammation and viral infection,^[^ ^59^, ^61^ ^]^ its specific role and activation mechanisms within neurotoxic astrocytes following ICH remain largely unexplored. Our study identifies the USP11‐PGAM5‐mtDNA‐cGAS‐STING axis as a novel and crucial intrinsic driver specifically within neurotoxic astrocytes, distinguishing it from the well‐documented microglial activation pathway.

Previous studies have established that mtDNA leakage into the cytosol involves mitochondrial outer membrane permeabilization,^[^ ^62^ ^]^ opening of the mPTP,^[^ ^63^ ^]^ and reactive oxygen species‐driven mechanisms,^[^ ^64^ ^]^ among others. mPTP opening is mainly triggered by oxidative stress and calcium overload.^[^ ^65^ ^]^ In our study, we found that mPTP promotes the accumulation of mtDNA in cytosol and triggers the activation of cGAS‐STING, subsequently leading to the activation of the downstream kinase TBK1. TBK1 phosphorylates and enhances the transcriptional activity of *Irf3* and NF‐κB, thereby initiating neurotoxic astrocyte reactivity. Furthermore, we also found PGAM5 promotes mitochondrial fission and mtDNA leakage through the induction Drp1S637 dephosphorylation. Consequently, PGAM5 regulates mPTP opening and Drp1‐dependent mitochondrial fission, which collectively trigger mitochondrial DNA leakage and cGAS‐STING activation.

These findings establish a critical link between the intrinsic mtDNA‐cGAS‐STING pathway and extrinsic immune remodeling. Specifically, PGAM5 causes mtDNA release and subsequent cGAS‐STING activation, thus promoting the production and release of CCL5.^[^ ^66^, ^67^, ^68^ ^]^ CCL5 mediates, through the CCL5‐CCR5 axis, the recruitment of peripheral neutrophils along with their subsequent NETosis. Thus, the astrocytic mtDNA‐cGAS‐STING pathway and neutrophil infiltration are functionally coupled, forming a coherent inflammatory cascade.

To increase the practical application and impact of our findings, we investigated the effects of pharmacological methods further. Targeting *Pgam5* with gene‐editing technology is effective for experiments, but its clinical application is restricted because the BBB limits drug penetration into the brain. Currently, multiple strategies (such as molecular Trojan horses, engineered nanoparticles, focused ultrasound‐mediated disruption, and stem cell‐based delivery) have been developed to enhance CNS drug delivery^[^ ^69^ ^]^; however, their therapeutic efficacy remains suboptimal due to issues including immunogenicity, limited targeting precision, and transient barrier. Thus, a highly effective system for delivering drugs to the brain is necessary. EVs offer unparalleled advantages for therapeutic delivery, including innate biocompatibility, the ability to traverse biological barriers like the BBB, intrinsic targeting capabilities, low immunogenicity, and versatile cargo‐loading capacity for diverse molecules.^[^ ^70^ ^]^ Surface modification of EVs incorporates diverse polypeptides (such as RVG, VCAM‐1), but the Ang2 peptide demonstrates superior receptor‐specific binding, significantly enhancing BBB penetration efficiency and precise astrocyte targeting.^[^ ^71^ ^]^ Consequently, considering the translational potential, a novel therapeutic strategy was developed utilizing engineered EVs modified by Ang2 for the targeted delivery of *Pgam5* siRNA to astrocytes. This brain‐targeted Ang2‐si*Pgam5*‐EVs system represents a significant innovation, as it overcomes the primary challenge of BBB penetration for siRNA therapeutics and achieves cell‐type‐specific targeting within the brain, which is a major hurdle in neurological drug development. Our results demonstrate that this approach significantly inhibits neurotoxic astrocyte reactivity and improves neurological deficits, presenting a promising and clinically relevant therapeutic strategy for ICH. Collectively, these findings not only identify the USP11–PGAM5–mtDNA–cGAS–STING axis as a key mediator of neurotoxic astrogliosis but also provide a novel, highly targeted delivery platform for its inhibition, thereby proposing a potential therapeutic avenue for ICH.

In conclusion, ICH triggers the conversion of astrocytes into the neurotoxic astrocytes phenotype, resulting in the release of neurotoxic factors such as complement components and inflammatory cytokines, which contribute to neuronal cell death. In order to mitigate secondary brain injury following ICH, it is imperative to impede the reactivity of neurotoxic astrocytes. In our study, we explained the function of the USP11‐PGAM5 axis in promoting mtDNA leakage through modulation of mPTP opening. This, in turn, activated the cGAS‐STING pathway, ultimately promoting the reactivity of neurotoxic astrocytes. Both in vivo and in vitro investigations demonstrated that targeting USP11‐PGAM5 axis or the cGAS‐STING pathway effectively prevented the activation of neurotoxic astrocytes, mitigated neuronal cell death, and facilitated neurological recovery. Collectively, these findings establish USP11‐PGAM5‐mtDNA‐cGAS‐STING pathway as a pivotal mediator of neurotoxic astrocyte reactivity and its inhibition presents a potential therapeutic avenue for ICH.

However, several limitations of our study should be acknowledged. Firstly, the current study focused specifically on the role of astrocytic PGAM5 during the acute and subacute phases following ICH, and did not investigate its role at the chronic phase. Future studies will include extended time points, such as six months and even one‐year post‐ICH, to evaluate the chronic effects of astrocytic PGAM5. Secondly, although our study supports a nonredundant role of the USP11‐PGAM5 axis in acute neurotoxic astrogliosis, we cannot fully exclude potential compensatory mechanisms mediated by other deubiquitinases or phosphatases over extended periods, which warrants further investigation in the future. Thirdly, the q‐PCR data indicated a higher level of *Ccl5* in microglia. However, immunofluorescence co‐staining revealed that CCL5 protein expression was rare in microglia. This discrepancy may result from post‐transcriptional or translational regulation in microglia, or from differences in protein turnover rates. Elucidating this mechanism will be a primary focus of our future study.

## 4 Experimental Section

### Human Brain Tissues

Brain tissue samples were collected from 12 patients undergoing surgery for ICH or brain tumors. For ICH cases, tissue adjacent to intracerebral hematomas was surgically removed per standard protocol to relieve focal pressure and prevent edema. This tissue was preserved for subsequent analysis. Control samples comprised histologically normal brain parenchyma resected during deep tumor access, intraoperatively verified as tumor‐free by neurosurgeons. Participants exhibiting autoimmune conditions, pre‐existing neurological diseases, CNS infections, or concurrent immunomodulatory therapies were excluded. The Ethics Committee of Tangdu Hospital, Fourth Military Medical University approved all procedures (K201906‐12). This study complied with Helsinki Declaration principles, with written informed consent obtained from patients or legal surrogates.

### Animals and Ethics

All experimental procedures were approved by the Ethics Committee of the Air Force Medical University, in accordance with the Animal Research: Reporting in Vivo Experiments (ARRIVE) guidelines. Male C57BL/6J mice (8‐12 weeks old) were obtained from the Animal Center of Air Force Medical University. Astrocyte‐specific *Pgam5* knockout mice were generated by crossing *Pgam5* floxed mice (Jackson Laboratory) with *Aldh1l1*‐CreERT2 mice (Jackson Laboratory). Tamoxifen (4 mg day^−1^; Selleck; S1972) was administered for five consecutive days. Animals were maintained in climate‐controlled housing with ad libitum access to food and water.

### ICH Mouse Model

To establish the ICH mouse model, a stereotaxic collagenase injection was performed.^[^ ^72^ ^]^ Briefly, the mice were anesthetized with 3% isoflurane and secured in a stereotactic apparatus. Subsequently, a 0.2 µL solution of 0.05 U collagenase (type VII, Sigma‐Aldrich, C0773) was stereotactically injected into the designated region (1.6 mm lateral and 1.0 mm anterior to the bregma, 3.4 mm deep) over a period of 5 min, followed by a 5‐min waiting period. In the sham group, mice underwent similar procedures, including needle insertion, but without collagenase injection. Moreover, the therapeutic potential of the CCL5‐CCR5 axis was evaluated by intraperitoneal administration of a CCL5‐neutralizing antibody (50 µg/mouse, R&D Systems, MAB478‐500) or a CCR5 antagonist (100 mg kg^−1^, Selleckchem, S2003).

### Neuroimaging

3‐T small‐animal magnetic resonance imaging (MRI) was utilized to determine the volumes of brain hemorrhage, following previously established protocols.^[^ ^73^ ^]^ Lesion volume quantification was performed using T2‐weighted imaging (T2) through summation of individual slice areas multiplied by the slice thickness. The volumes were meticulously delineated and computed using ImageJ software. Two independent investigators, who were unaware of the experimental grouping, meticulously analyzed and computed the data.

### BBB Permeability

BBB integrity was assessed via Evans blue extravasation 72 h post‐ICH induction. Three h prior to euthanasia, mice were intravenously administered Evans blue (4 mL kg^−1^, Sigma‐Aldrich, E2129) through the tail vein. Under deep anesthesia induced by 2% sodium pentobarbital (lethal dosage), subjects received transcardial perfusion with 40 mL ice‐cold physiological saline. After tissue weighing, brains were sectioned coronally into six uniform slices for macroscopic analysis. Homogenization in saline preceded centrifugation (12 000 × *g*, 30 min, 4 °C). Resulting supernatants were mixed 1:1 with trichloroacetic acid and incubated overnight at 4 °C. Following secondary centrifugation (identical parameters), Evans blue levels were measured spectrophotometrically at 620 nm.

### Brain Water Content

Upon sacrifice of the mice, the harvested brain was partitioned into three sections: the ipsilateral hemisphere, the contralateral hemisphere, and the cerebellum. Each section was promptly weighed to obtain the wet weight. Subsequently, these tissues were subjected to drying at 100 °C for a duration of one day to obtain the dry weight. The brain water content was then calculated using the following formula: (wet weight – dry weight)/wet weight × 100%.

### Neurobehavioral Tests

Adhesive removal test. A 2×3 mm adhesive tape segment was affixed to the left forepaw (impaired limb). Latencies to initial oral contact and complete tape removal were recorded within a 120‐s observation window.

Rotarod test. Animals underwent three daily trials on an accelerating rod (4–40 rpm over 5 min), separated by 5‐min intervals. Mean fall latency was calculated from triplicate measurements.

Morris Water Maze (MWM). MWM test was performed to determine the Spatial cognitive function. On postoperative days 16–20 following ICH, mice received three daily training sessions (with 30‐min intervals) in a circular pool containing opaque water maintained at ambient temperature. During each trial, mice were randomly released from one of four quadrants. Navigation trajectories to the submerged platform were recorded over a 60‐s period to evaluate spatial learning capacity. Mice that failed to reach the platform within the allotted time were gently placed on it for a 10‐s retention period. On day 21, a probe trial was conducted by removing the platform and releasing mice from the quadrant opposed to the original platform location for 60 s of free exploration. During the 60 s trial, including the percentage of time spent in the target quadrant and the frequency of platform crossings, were recorded and calculated.

### TUNEL Assay

Cellular apoptosis was assessed using TUNEL staining (Elabscience, E‐CK‐A321). Following 6‐h fixation in 4% paraformaldehyde, tissues were cryosectioned, then permeabilized with 0.5% Triton X‐100/PBS for 30 min at room temperature. Tissue sections were incubated with TUNEL reagent (37 °C, 60 min) followed by DAPI (Beyotime, P0131) counterstaining (37 °C, 10 min). The apoptotic index was quantified as the ratio of TUNEL‐positive to DAPI‐labeled nuclei.

### Western Blotting Analysis

The tissues were homogenized in RIPA lysis buffer (Beyotime, P0013B) supplemented with protease inhibitor for 30 min. Following centrifugation at 12000 × *g* for 15 min, the supernatant was collected. The protein concentration was determined using a BCA protein assay kit (Thermo Fisher Scientific, A55864). Subsequently, protein samples were loaded onto SDS‐PAGE gels and transferred onto PVDF membranes. The membranes were then blocked with 5% milk for 1 h at room temperature. Next, the membranes were incubated overnight with primary antibodies, including anti‐PGAM5 (1:1000; Abcam; ab126534), anti‐USP11 (Abcam; ab109232), anti‐ubiquitin (Proteintech, 10201‐2‐AP), anti‐Claudin5 (Proteintech, 29767‐1‐AP), anti‐Occludin (Proteintech, 27260‐1‐AP), anti‐Bax (Proteintech, 50599‐2‐Ig), anti‐Bcl2 (Proteintech, 26593‐1‐AP), anti‐cGAS ((1:2000; Novus; NBP3‐16666), anti‐STING (1:1000; Cell Signaling Technology; D2P2F), anti‐p‐TBK1 (1:1000; Cell Signaling Technology; D52C2), anti‐TBK1 (1:1000; Cell Signaling Technology; D1B4), anti‐NF‐κB (1:1000; Proteintech; 80979‐1‐RR), anti‐p‐NF‐κB (1:1000; Cell Signaling Technology; 93H1), anti‐IRF3 (1:5000; Proteintech; 11312‐1‐AP), anti‐p‐IRF3 (1:5000; Cell Signaling Technology; D6O1M), anti‐DRP1 (1:5000; Proteintech; 12957‐1‐AP), anti‐p‐DRP1(Ser616) (1:1000; Cell Signaling Technology; D9A1), anti‐p‐DRP1(Ser637) (1:2000; Cell Signaling Technology; F8V7T), anti‐TFAM (1:5000; Cell Signaling Technology; F5A3U), anti‐CD63 (1:1000; Proteintech; 32151‐1‐AP), anti‐CD81 (1:1000; Proteintech; 27855‐1‐AP), anti‐TSG101 (1:5000; Proteintech; 28283‐1‐AP), and anti‐GM130 (1:5000; Invitrogen; MA5‐35107) as well as anti‐β‐actin (1:5000; Cell Signaling Technology; 13E5) as a loading control. After washing the membranes with TBST, they were incubated with secondary antibodies for 1 h at room temperature. After additional TBST washes, protein bands were detected via Bio‐Rad ChemiDoc imaging and quantified using ImageJ software.

### Immunofluorescence Staining

Following humane euthanasia, mice underwent transcardial perfusion with 30 mL PBS followed by 4% PFA. The brain was carefully extracted and immersed in a solution of 4% paraformaldehyde for a period of 8 h. Following this fixation step, the brain tissue was dehydrated using a 30% sucrose solution, allowing for optimal preservation. The dehydrated brain tissue was then sliced into 25 µm coronal sections. The sections were incubated for 15 min in a solution containing 0.3% Triton X‐100, facilitating cell permeabilization. Subsequently, the sections underwent a 30‐min incubation with 10% donkey serum to block nonspecific binding sites. Immunostaining was then carried out by incubating the sections overnight at 4 °C with primary antibodies: anti‐GFAP (1:1000; Invitrogen; PA1‐10004), anti‐PGAM5 (1:300; Abcam; ab126534), anti‐C3 (1:200; Abcam; ab97462), anti‐Neun (1:500; Merck; ABN90), anti‐MAP2 (1:200; Abcam; ab5392), anti‐IBA1 (1:300; Novus; NB100‐1028), anti‐CD86 (1:300; Proteintech; 13395‐1‐AP), anti‐RANTES (1:200; Abcam; ab322195), anti‐Histone H3 (1:500; Abcam; ab300641), and anti‐Ly6G (1:200; Proteintech; 65078‐1‐IG). Following the primary antibody incubation, the sections were thoroughly washed with PBS to remove any unbound antibodies. Next, the sections were exposed to correspondingly secondary antibodies at a temperature of 37 °C for 1 h. Finally, the stained sections were imaged using an A1 Si confocal microscope (Nikon), allowing for high‐resolution visualization and analysis of the protein distribution within the brain tissue.

### Flow Cytometry Analysis

Mice were euthanized via cervical dislocation at day 3 after ICH, with brains immediately harvested and immersed in ice‐cold Hank's Balanced Salt Solution (HBSS). Tissue digestion employed 2 mg mL^−1^ Papain/HBSS at 37 °C for 60 min under continuous gentle rotation. The resultant cell suspension was filtered through 50‐µm nylon mesh before centrifugation (300 × g, 5 min, 4 °C). For immunophenotyping, cells were labeled with fluorochrome‐conjugated mAbs: APC‐Cy7 anti‐mouse CD45 (Thermo, A15395), Pacific Blue anti‐mouse Ly6C (Biolegend, 128064), PE anti‐mouse Ly6G (Thermo, 16‐9668‐82), PE‐Cy7 anti‐mouse CD3e (Thermo, 25‐0031‐82), FITC anti‐mouse CD4 (Thermo, 11‐0041‐86), and APC anti‐mouse CD8 (Thermo, 17‐0081‐82). Samples were analyzed on a NovoCyte 3000 flow cytometer, with data processed using NovoExpress Software (v1.4.1).

### LC‐MS/MS Analysis

Proteins isolated through PGAM5 immunoprecipitation underwent LC‐MS/MS analysis, utilizing IgG‐immunoprecipitated specimens as controls. After silver staining validation on 10% SDS‐PAGE gels, excised bands were subjected to proteomic characterization by PTM Biolabs, Inc. (Hangzhou, China).

### Coimmunoprecipitation

Cell lysis was performed for 5 h at 4 °C using IP buffer (Thermo Scientific, 87 787) containing protease inhibitor cocktail (1:100) with gentle rotation. Following centrifugation (12 000 × *g*, 15 min, 4 °C), 1 mg aliquots of clarified supernatant were subjected to overnight incubation (14 h, 4 °C) with antibody‐conjugated protein A/G magnetic beads (ACE Biotechnology, BK0004‐02). Primary antibodies included: anti‐ubiquitin (Proteintech, 10201‐2‐AP), anti‐PGAM5 (Abcam, ab126534), and anti‐USP11 (Abcam, ab109232), with constant end‐over‐end rotation.

### Cell Culture and Treatment

The primary astrocytes were cultured following previously described methods. Briefly, neonatal C57Bl/6 mice were sacrificed, and their brain cortices were excised and transferred to ice‐cold Hank's solution lacking magnesium and calcium. The dissociated cells were then suspended in Dulbecco's modified Eagle's medium (DMEM) supplemented with 100 U/ml streptomycin, 100 mg mL^−1^ penicillin, and 10% fetal bovine serum (FBS). The cells were allowed to proliferate for 10 days in a humidified incubator with 5% CO2 and 95% air following enzymatic digestion. Subsequently, the astrocytes in T75 flasks were agitated at 220 rpm for 12 h to isolate purified astrocytes. Primary neuronal cultures were established from embryonic day 18 C57BL/6 mice. Cerebral cortices were isolated, finely minced, and enzymatically digested using trypsin. Dissociated neurons were seeded onto poly‐L‐ornithine/laminin‐coated dishes (Sigma‐Aldrich, LPLO001) and cultured in Neurobasal Medium (Gibco, 21103 049) containing L‐glutamine (Solarbio, G0200), B27 supplement (Gibco, 17504044), and penicillin/streptomycin (Beyotime, C0223) for 7 days. Primary astrocytes were stimulated with a cytokine cocktail comprising IL‐1α (3 ng mL^−1^, 400‐ML‐005, R&D), TNF‐α (30 ng mL^−1^, 210‐TA ‐005, R&D), and C1q (400 ng mL^−1^, MyBioSource, MBS143105) for 24 h to induce a neurotoxic phenotype. For the cell co‐culture experiments, primary astrocytes were initially grouped and treated with ITC, PGAM5‐OV, or PGAM‐NC. Following treatment, the culture medium was replaced with fresh medium. The impact of astrocyte‐secreted toxic factors in this fresh medium on the survival of primary neurons was then assessed. In one approach, primary neurons were directly seeded onto the culture dish containing the treated primary astrocytes and observed. In the other approach, conditioned medium from the treated primary astrocytes was transferred to primary neurons for observation. For the blocking antibody experiment, primary microglia were treated with anti‐CCL5 (2 µg mL^−1^, R&D Systems, MAB478‐500) concurrently with the CM, while in another group, primary microglia were pretreated with a CCR5 antagonist (500 nmol/L, Selleckchem, S2003) for 1 h prior to the addition of CM. All cells were maintained in a humidified incubator at 37 °C with 5% CO2 and 95% atmospheric air. Plasmid and siRNA transfections employed Lipofectamine 2000 Reagent (Invitrogen, Carlsbad, CA, USA) per manufacturer's instructions.^[^ ^74^ ^]^ Specific gene primer sequences are listed in Table S1 (Supporting Information).

### RNA‐seq

Following the isolation of primary astrocytes as previously described, the cells were treated with ITC and subsequently transfected with either PGAM5‐OV adenovirus or control adenovirus. Cells were then lysed using TRIzol reagent, and the lysates were gathered and frozen at ‐80 °C until they could be processed for sequencing.

### Quantitative real‐time PCR (qRT‐PCR)

First, total RNA was extracted using TRIzol Reagent (Invitrogen, USA, 15596018CN) following standardized protocols. Quantitative PCR (qPCR) analysis was conducted on a Bio‐Rad Real‐Time PCR System. The measurement of cytosolic mtDNA content was conducted in accordance with previously described methods. Primary astrocytes were collected and divided into two equal portions for the isolation of cytosolic and total DNA. Cell fractions were prepared using the Cell Fractionation Kit (Abcam, ab109719) following the manufacturer's instructions. Cytosolic and whole‐cell DNA were extracted using QIAquick Nucleotide Removal Columns (Qiagen, 28115) and quantified using qPCR. The primer sequences used for qPCR are provided in Table S2 (Supporting Information).

### Enzyme‐Linked Immunosorbent Assays (ELISA)

CXCL16, CXCL10, CCL7, CXCL1, CXCL9, CCL2, CCL5, and CCL25 levels were quantified using a commercial ELISA kit (CXCL16: Abcam, ab197744; CXCL10: Abcam, ab9807; CCL7: Invitrogen, BMS6006INST; CXCL1: Biolegend, 447504; CXCL9: Abcam, ab242976; CCL2, Invitrogen, 88‐7391‐22; CCL5: Abcam, ab100739; CCL25: Invitrogen, EMCCL25) following the manufacturer's instructions. Briefly, capture antibodies (100 µL well^−1^) were immobilized on 96‐well plates via overnight incubation at 4 °C. After blocking with 1X ELISA diluent (200 µL well^−1^, 1 h), standard curves were generated through serial dilution of recombinant proteins. Detection antibodies (100 µL well^−1^) were subsequently administered followed by avidin‐HRP conjugate incubation (30 min). Optical density at 450 nm was measured using a microplate reader.

### Assessment of Mitochondrial Permeability Transition Pore (mPTP) Opening

To assess the opening of the mPTP, the mPTP Assay Kit (C2009S, Beyotime) was employed following the manufacturer's protocol. Briefly, cells were treated with 1 ×10^−3^ m calcein‐AM and 5 ×10^−3^ m cobalt chloride (CoCl2) in darkness at 37 °C for 30 min. After incubation, fresh culture medium was added to the cell culture and further incubated in the dark at 37 °C for an additional 30 min to ensure complete hydrolysis of calcein‐AM by endonucleases, resulting in the production of green fluorescent calcein. The cells were then washed three times with PBS before being evaluated using a confocal microscope.

### Isolation and Characterization of EVs

EVs were isolated from serum‐free Opti‐MEM (Umibio, Shanghai, China, 31985062) following 24‐ho incubation. Conditioned medium was supplemented with protease inhibitors prior to sequential centrifugation: 300 × *g* (5 min, 4 °C), 2000 × *g* (10 min, 4 °C), and 10 000 × *g* (30 min, 4 °C). Clarified supernatants were filtered through 0.22‐µm filters to eliminate cellular debris. Filtered fractions underwent ultracentrifugation at 100 000 × *g* for 70 min. After supernatant removal, EV pellets were stored at 20 °C for subsequent analysis. To observe their morphology, the isolated EVs underwent preparation for TEM imaging, and their size and concentration were assessed by resuspending them in filtered PBS and using a ZetaView instrument (Particle Metrix, Germany). EV protein marker expression profiles and negative control markers were assessed by western blot analysis.

### Loading of Ang2‐EVs with siPgam5 Followed by Intravenous Injection

HEK293T cells were maintained at 37 °C/5% CO_2_. At 70–80% confluency, transfection with Ang2‐Lamp2b plasmid was performed using Lipofectamine 2000 per manufacturer's protocol. Medium was replaced with EV‐depleted solution 24 h post‐transfection. Conditioned medium was harvested, with Ang2‐EVs isolated from plasmid‐transfected HEK293T supernatants via sequential centrifugation. Control simulated EVs (Mock‐EVs) was done using cells that had not been transfected. Following supernatant removal, EV pellets were stored at 20 °C for subsequent use. si*Pgam5* or negative control was electroporated into Ang2‐EVs. siRNA sequences appear Table S1 (Supporting Information). Brain‐injured mice received intravenous Ang2‐si*Pgam5*‐EVs or mock‐si*Pgam5*‐EVs starting 1 h post‐injury, administered every 24 h for 12 days.

### EV Labeling and Tracking In Vivo

Mock‐EVs and Ang2‐EVs were labeled with DiR (Yeasen, 40757ES25) via 40‐min room temperature incubation. Free dye was eliminated by ultracentrifugation (100 000 ×*g*, 70 min, 4 °C). Labeled EVs (200 µg) were intravenously injected through the tail vein. Whole‐body biodistribution and organ‐specific localization were evaluated 24 h post‐injection using an IVIS Spectrum imaging system (PerkinElmer, USA). For histological analysis, DiR‐labeled Ang2‐si*Pgam5*‐EVs were incubated for 30 min at room temperature (RT), then intravenously administered via tail vein injection. Fresh brain tissue was harvested, rinsed, and OCT‐embedded. Sections blocked with 5% BSA for 1 h were immunostained with anti‐GFAP antibody (Invitrogen, PA1‐10004; 1:1000) overnight at 4 °C. After PBS washes, Alexa Fluor 488‐conjugated donkey anti‐chicken secondary antibody (Invitrogen; 1:500, A78948) was applied for 2 h at RT in darkness. Nuclei were counterstained with DAPI. Confocal images acquired on a Nikon A1Si system were quantified using ImageJ.

### Statistics

Statistical analyses were performed using GraphPad Prism 9.0. Data are expressed as mean ± SD unless specified. Continuous normally distributed variables between two groups were compared using two‐tailed Student's *t*‐tests. For multigroup comparisons, one‐way or two‐way ANOVA with Bonferroni post hoc testing was employed. All procedures from group assignment through data collection to analysis were conducted under blinding. Statistical significance was defined as *p* < 0.05.

## Conflict of interest

The authors declare no conflicts of interest.

## Author Contributions

J.H., Z.Q., and Y.C. contributed equally to this work. Designed the study: W.G., Y.Q., X.W; wrote the manuscript: J.H.; revised the manuscript: W.G., Y.Q., X.W; performed animal studies: J.H., Q.H; performed the in vitro experiments: J.H., T.W; performed the cell experiments: Z.Q., Q.H., T.W., P.Y; analyzed the clinical data: Y.C., Y.X; analyzed RNA‐seq datas: Z.Q., Y.C; typeset figures: J.H; all authors approved the final report.

## Supporting information



Supporting Information

## Data Availability

The data that support the findings of this study are available from the corresponding author upon reasonable request.
