# Supplementary material for: USP11‐PGAM5 Axis Promotes Neurotoxic Astrocyte Reactivity by Aggravating the mtDNA‐cGAS‐STING Pathway After Intracerebral Hemorrhage
Source: Adv Sci (Weinh). 2025 Nov 7;13(1):e14283. doi: 10.1002/advs.202514283 (PMC12767119; doi:10.1002/advs.202514283)
Supplement: Supplementary file 1 — Supporting Information [file ADVS-13-e14283-s001.docx]

**Supplementary Materials for**

**USP11-PGAM5 Axis Promotes Neurotoxic Astrocyte Reactivity by Aggravating the mtDNA-cGAS-STING Pathway after Intracerebral Hemorrhage**

**The file includes:**

**Figures. S1 to S7**

**Tables. S1 to S2**

**
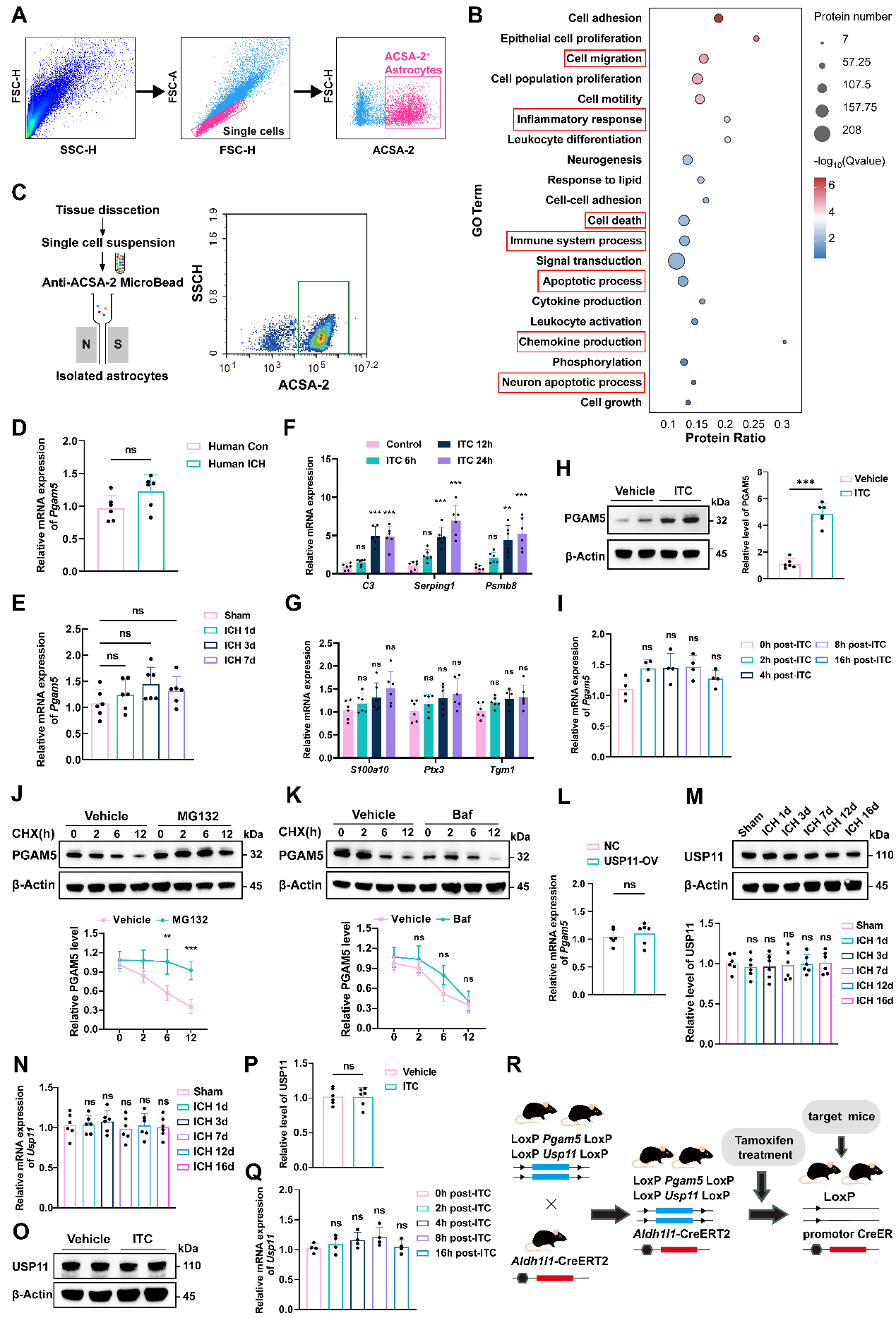
**

**Figure S1. PGAM5 elevation was independent of transcriptional regulation in both ICH patients and ICH mice. (A)** Isolation of astrocytes through flow cytometry. **(B)** GO analysis following proteomic sequencing. **(C)** Isolation of astrocytes through anti-ACSA-2 magnetic beads. **(D-E)** qPCR analysis of *Pgam5* mRNA in astrocytes isolated from ICH patients and ICH mice (n = 6 per group, one-way ANOVA). **(F-G)** PCR analysis of mRNA levels of astrocyte A1 markers, including *C3*, *Serping1*, and *Psmb8*, and astrocyte A2 markers, including *Ptx3*, *S100a10*, and *Tgm1*, at different time points after ITC treatment (n = 6 per group, one-way ANOVA). ITC treatment: IL-1α, TNF-α, and C1q (ITC) treatment. **(H)** Western blot analysis of PGAM5 protein expression with or without ITC treatment (n = 6 per group, Student’s t-test). **(I)** qPCR analysis of *Pgam5* mRNA expression was performed in primary astrocytes with or without ITC treatment (n = 4 per group, one-way ANOVA). **(J-K)** Western blot analysis and quantification of PGAM5 expression in CHX-treated primary astrocytes following the treatment of MG132 or Baf (n = 3 per group, two-way ANOVA). **(L)** The mRNA level of *Pgam5* in HEK293T cells with or without USP11 overexpression (n = 6 per group, Student’s t-test). **(M)** Western blot analysis was conducted on USP11 in isolated astrocytes at various time intervals after ICH surgery in mice (n = 6 per group, one-way ANOVA). **(N)** qPCR analysis of *Usp11* mRNA in astrocytes isolated from ICH mice (n = 6 per group, one-way ANOVA). **(O-P)** Western blot analysis of USP11 protein expression with or without ITC treatment (n = 6 per group, Student’s t-test). **(Q)** qPCR analysis of *Usp11* mRNA expression was performed in primary astrocytes with or without ITC treatment (n = 4 per group, one-way ANOVA). **(R)** Construction of astrocyte-specific *Pgam5* or *Usp11* knockout mice. Data are presented as means ± SD. *p < 0.05, **p < 0.01, ***p < 0.001.


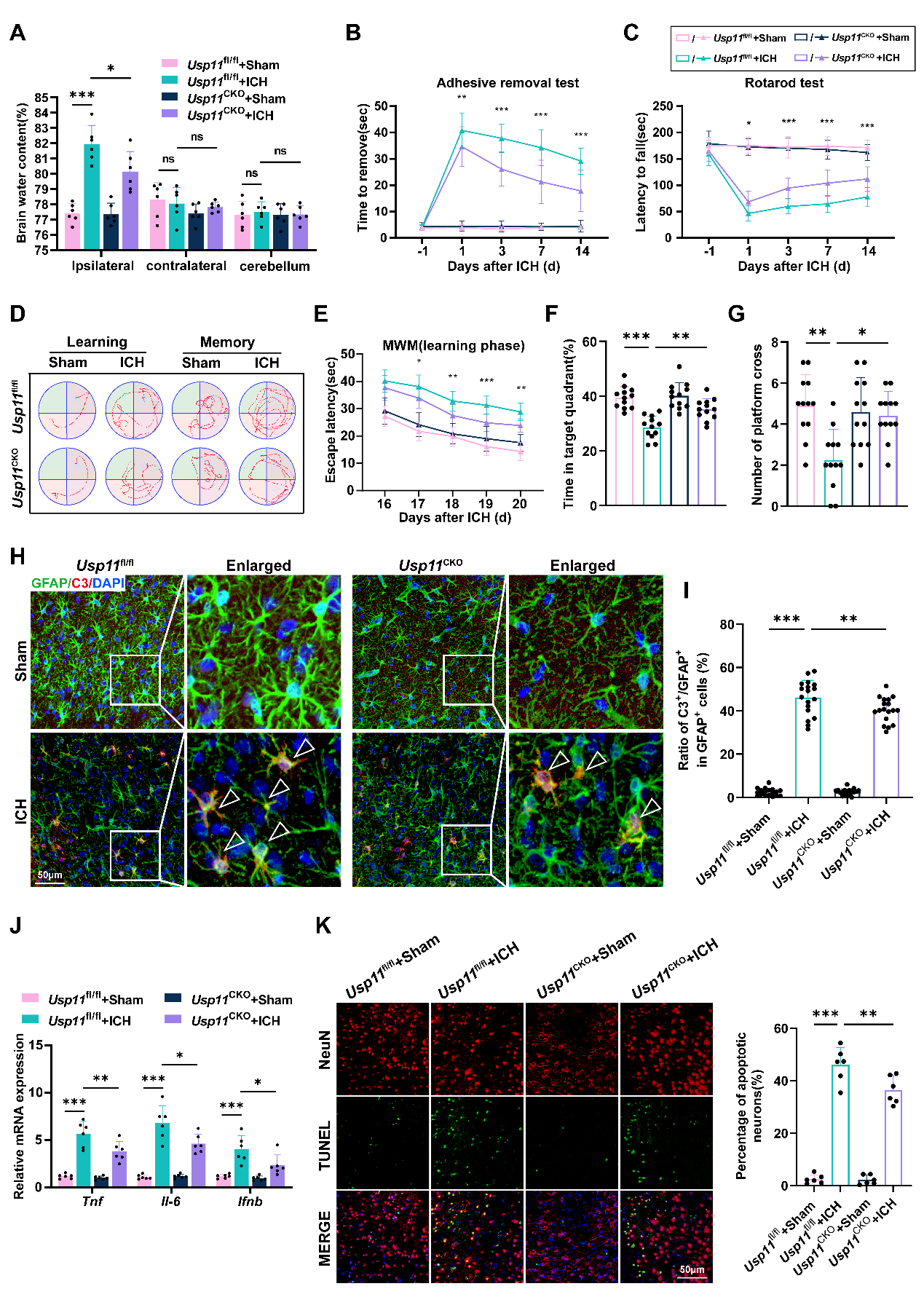


**Figure S2. Conditional deletion of *Usp11* in astrocytes promotes neurological recovery, inhibits neurotoxic astrocyte reactivity and reduces neuronal apoptosis after ICH.** **(A)** Brain edema was assessed via water content measurement at 72 hours post-ICH (n = 6 per group, one-way ANOVA). **(B-C)** Sensorimotor function was evaluated using adhesive removal **(B)** and rotarod behavioral assays **(C)** (n = 12 per group, two-way ANOVA). **(D-G)** Cognitive performance was evaluated through Morris Water Maze (MWM) testing, assessing spatial learning and memory (n = 12 per group, E for two-way ANOVA, F for one-way ANOVA, G for Kruskal-Wallis H test). **(H-I)** On day 3 after ICH, brain sections were processed for dual immunofluorescence labeling for GFAP and C3, followed by quantification of C3⁺ cells within GFAP⁺ astrocytes (n = 18 slices from 6 mice per group, one-way ANOVA). Scale bar: 50 μm. **(J)** Quantitative PCR measured transcript levels of key inflammatory genes (*Tnf*, *Il6*, *Ifnb*) in isolated astrocytes at 72 h post-ICH (n = 6 per group, one-way ANOVA). **(K)** Representative TUNEL staining images across experimental groups and the quantification of apoptotic cells at 72 h post-ICH (n = 6 per group, one-way ANOVA). Scale bar: 50 μm. Data are presented as means ± SD. *p < 0.05, **p < 0.01, ***p < 0.001. Behavioral analyses: Asterisks indicate daily comparisons betwee the two groups (ICH-*Usp11*^flox/flox^ group vs ICH-*Usp11*^CKO^ group).


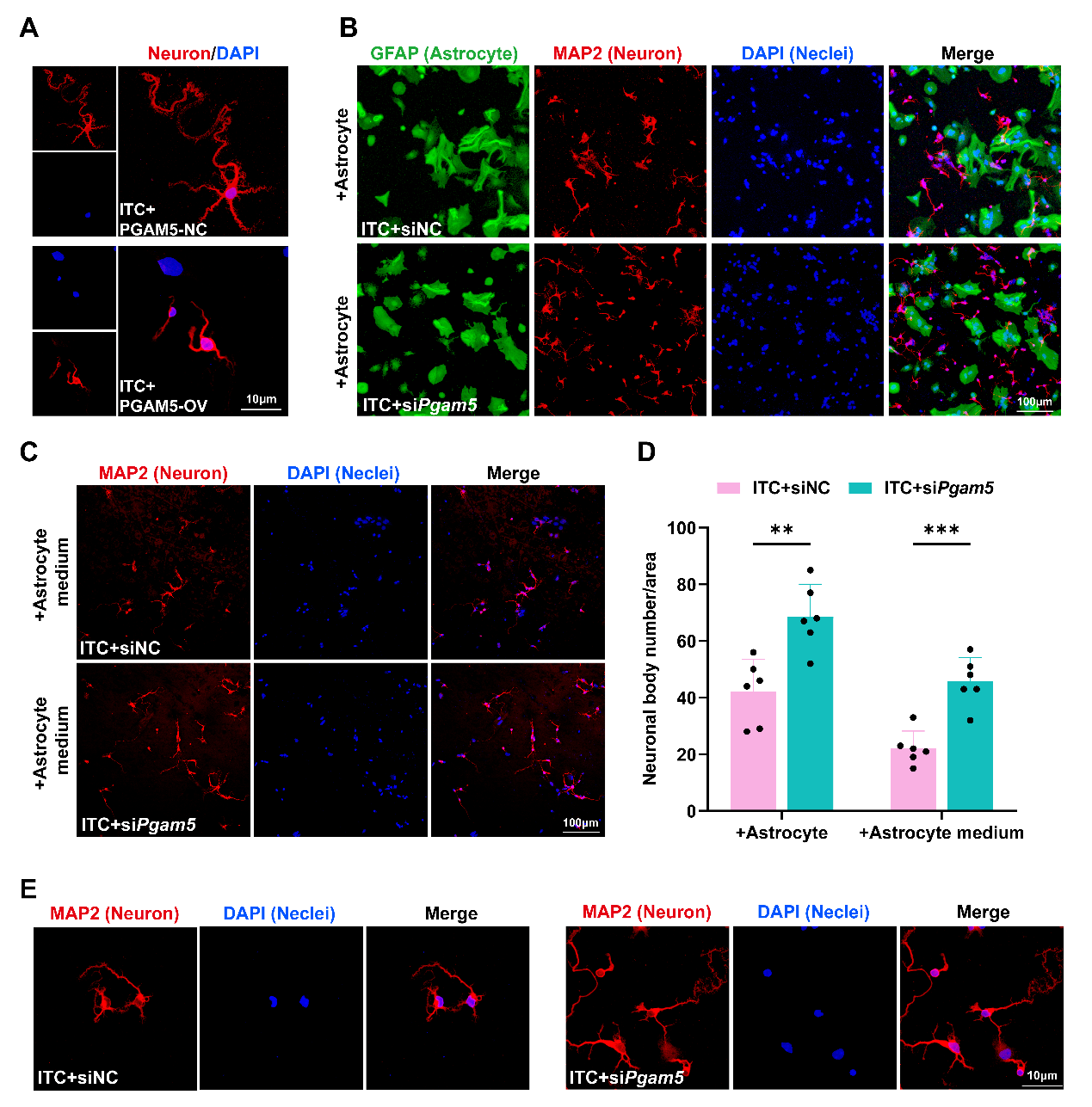
**Figure S3. Effects of PGAM5-OV or si*Pgam5* co-culture on neuronal toxicity. (A)** Representative immunofluorescence images analyzing primary neuronal morphology post-co-culture in each group. Scale bars:10 μm. **(B-D)** Representative images and the quantification of the number of neuronal cell bodies following co-culture with siNC or si*Pgam5* primary astrocytes, or treatment with their conditioned medium (n = 6 per group, one-way ANOVA). Scale bars:100 μm. **(E)** Characteristic immunofluorescence micrographs illustrating neuronal morphology following co-culture across experimental conditions. Scale bars:10 μm. Data are presented as means ± SD. *p < 0.05, **p < 0.01, ***p < 0.001.

**
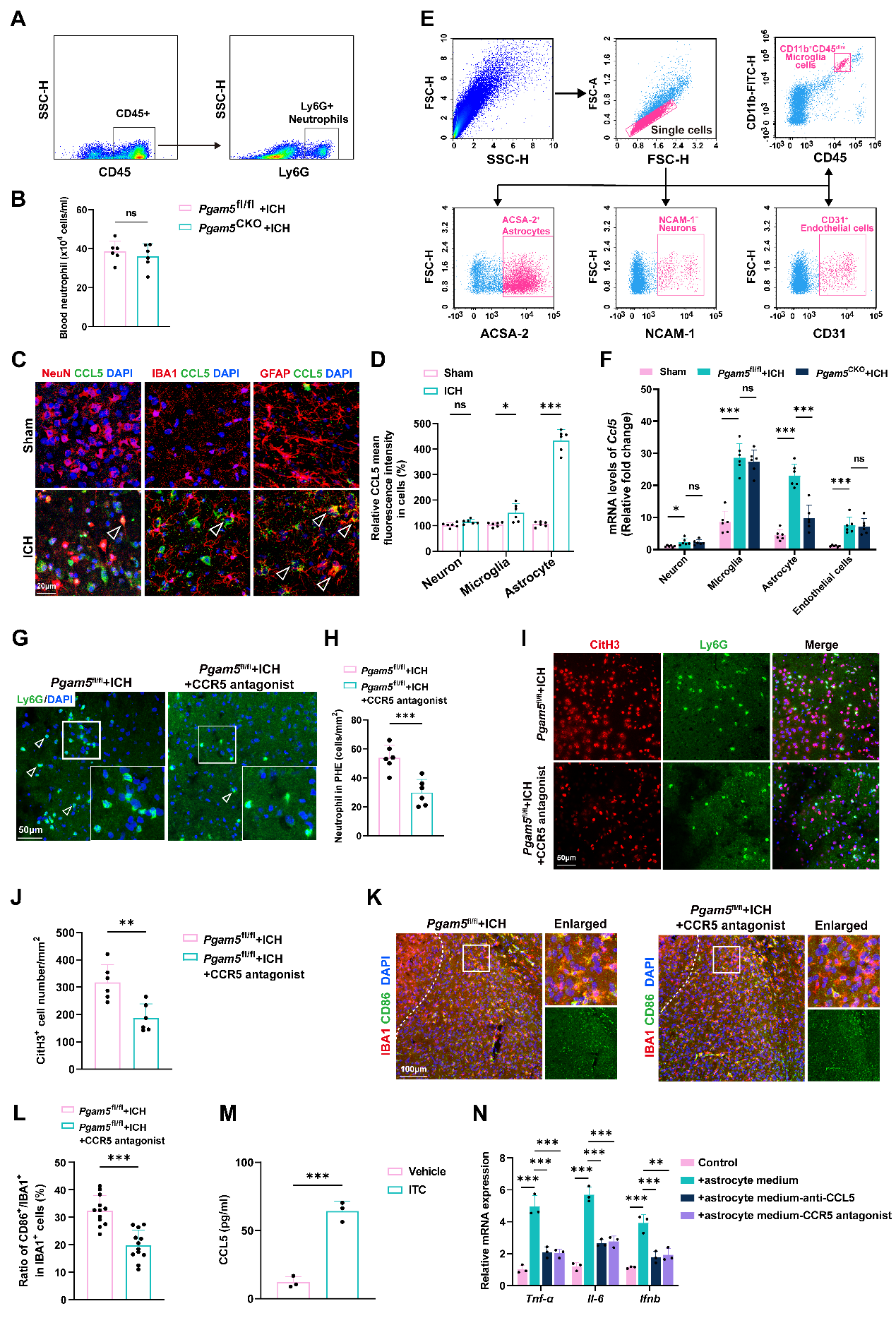
Figure S4. Astrocyte-specific *Pgam5* deletion reduced neutrophil infiltration via the CCL5-CCR5 signaling axis.** **(A-B)** Flow cytometric analysis of the cell numbers of CD45+Ly6G+ neutrophils in blood of *Pgam5*^fl/fl^ mice or *Pgam5*^CKO^ mice post ICH (n = 6 per group, Student’s t-test). **(C-D)** Representative immunostaining images and statistical analysis of CCL5 expression in neurons (NeuN⁺ cells), microglia (IBA1⁺ cells), and astrocytes (GFAP⁺ cells) in brain tissue from ICH mice and controls (n = 6 per group, Student’s t-test). Scale bar:20 μm. **(E)** Schematic of neurons, astrocytes, microglia, and endothelial cells isolation from ICH mice. **(F)** Relative expression of *Ccl5* in the sorted cells as determined by qPCR (n = 6 per group, one-way ANOVA). **(G-H)** Immunofluorescence targeting Ly6G (green) was conducted using brain sections from *Pgam5*^flox/flox^ ICH mice with or without CCR5 antagonist (n = 6 per group, Student’s t-test). Scale bar:50 μm. **(I-J)** Quantification analysis of the CitH3^+^ cell number per mm^2^ following CCR5 antagonist treatment in the ICH model (n = 6 per group, Student’s t-test). Scale bar:50 μm. **(K-L)** Representative micrographs of IBA1 and CD86 expression, along with quantitative analysis of CD86+ cells in IBA1+ cells following CCR5 antagonist treatment in the ICH model (n = 12 slices from 6 mice per group, Student’s t-test). Scale bar:100 μm. Hemorrhage borders are demarcated by distinct dashed lines. **(M)** CCL5 levels in the culture medium of ITC-treated primary astrocytes were measured using ELISA (n = 3 per group, Student’s t-test). **(N)** Quantitative PCR was performed to measure the transcript levels of key inflammatory genes (*Tnf*, *Il6*, *Ifnb*) in primary microglia under various treatment conditions (n = 3 per group, one-way ANOVA). Data are presented as means ± SD. *p < 0.05, **p < 0.01, ***p < 0.001.


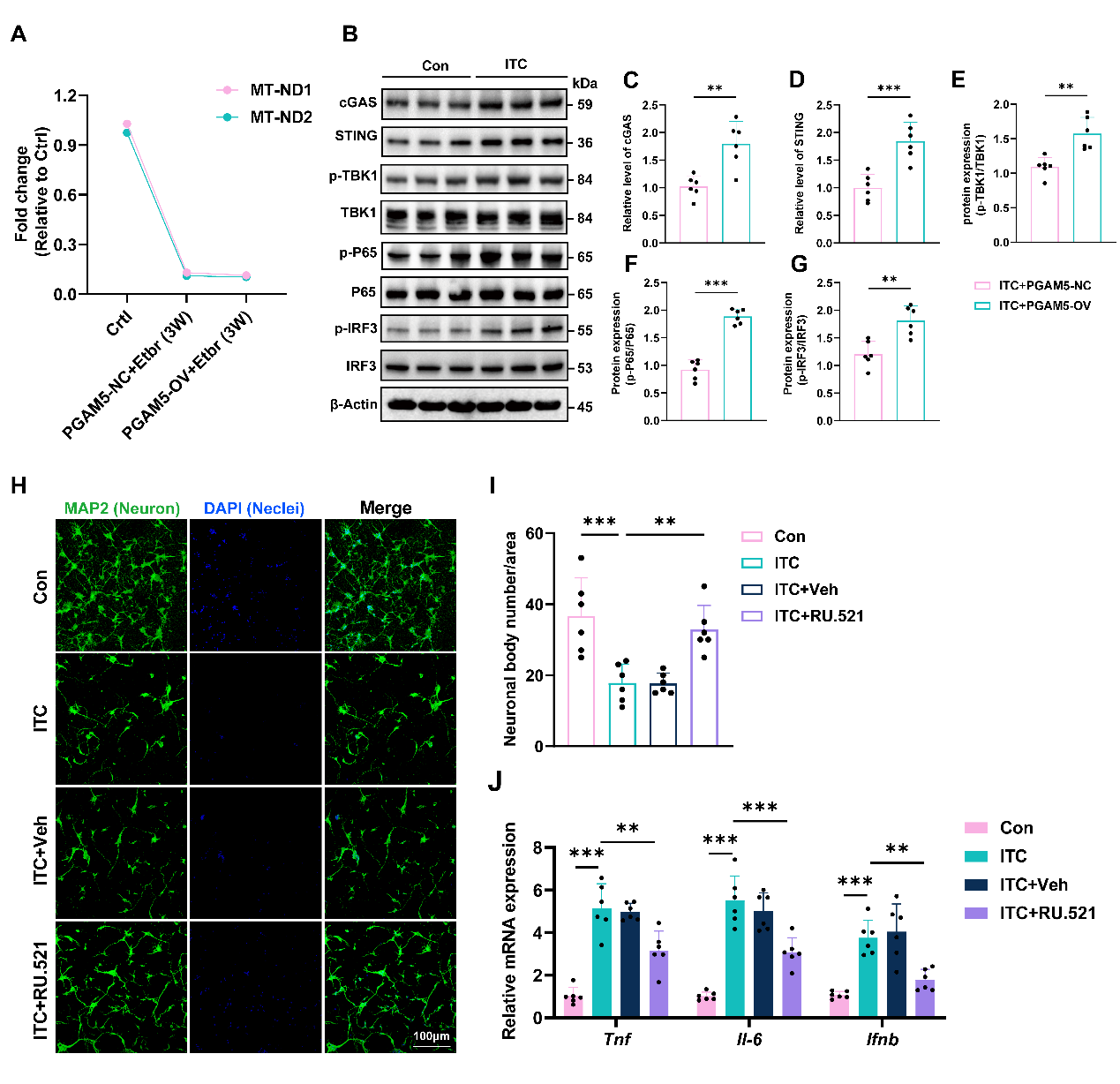


**Figure S5. PGAM5 activates the cGAS-STING pathway by triggering mitochondrial DNA release. (A)** Representative qPCR analysis of mtDNA depletion from primary astrocytes over three weeks treatment in EtBr. (n = 3 per group, two-way ANOVA). **(B)** Western blot analysis of the expression level of cGAS-STING and several downstream targets in ITC treated astrocytes. **(C-G)** Quantification of the expression of cGAS, STING, p-TBK1/TBK1, p-P65/P65 and p-IRF3/IRE3 in each group (n = 6 per group, Student’s t-test). **(H-I)** Representative images and the quantification of the number of neuronal cell bodies following treatment with ITC or RU.521 (n = 6 per group, one-way ANOVA). Scale bars:100 μm. **(J)** PCR analysis of mRNA level of several inflammatory cytokines including *Tnf*, *Il6* and *Ifnb* in ITC treated astrocytes with or without treated with RU.521 (n = 6 per group, one-way ANOVA). Data are presented as means ± SD. *p < 0.05, **p < 0.01, ***p < 0.001.


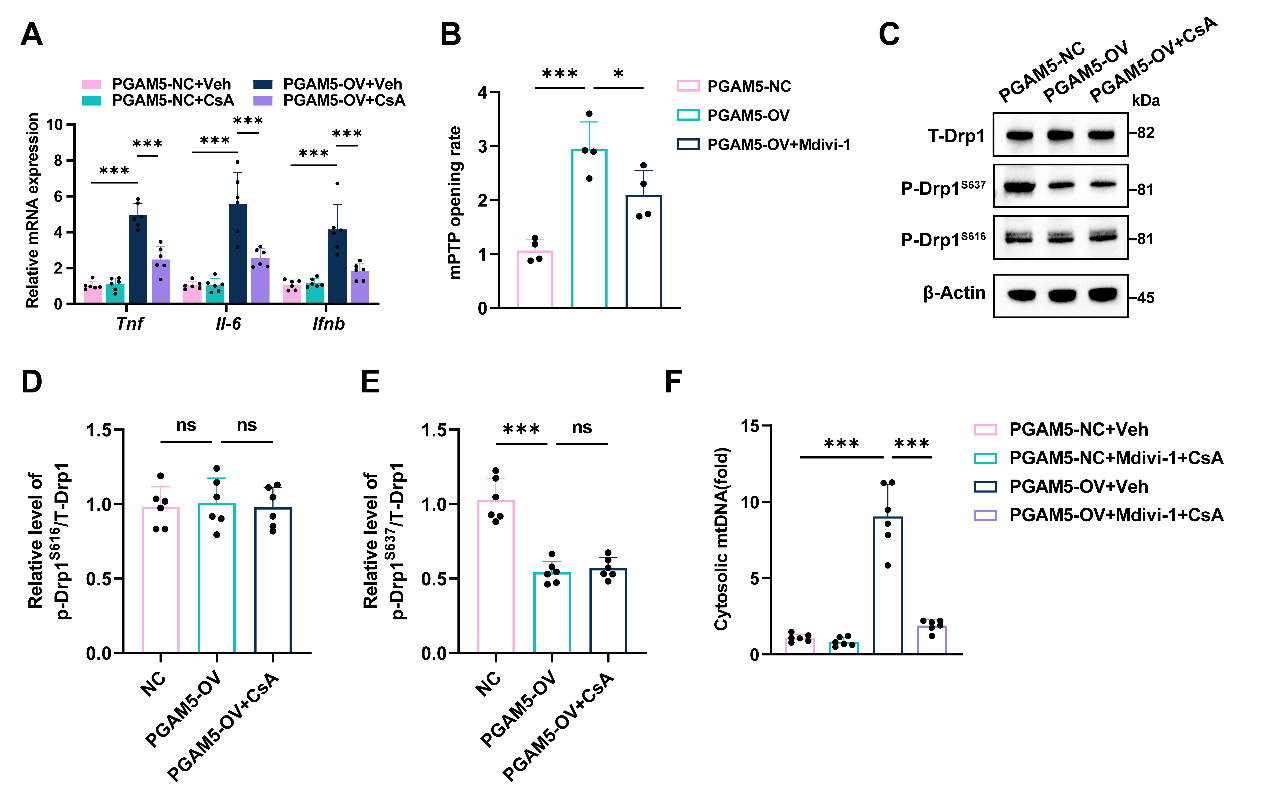


**Figure S6. Drp1 activation promotes mPTP opening whereas mPTP inhibition does not affect Drp1 dephosphorylation and combined inhibition of both pathways yields superior protective effects against mtDNA leakage.** **(A)** The effects of pharmacological inactivation of the mPTP using cyclosporin A (CsA) on PGAM5 induced production of inflammatory cytokines including *Tnf*, *Il6* and *Ifnb* (n = 6 per group, one-way ANOVA). **(B)** Quantification of mPTP opening rate in cultured PGAM5-OV primary astrocytes with or without Mdivi-1 treatment (n = 4 per group, one-way ANOVA). **(C-E)** Western blot assay of the effects of CsA on the expression of Drp1, p-Drp1^S637^ and p-Drp1^S616^ and the related statistical analysis (n = 6 per group, one-way ANOVA). **(F)** Combined treatment with Mdivi-1 and CsA synergistically attenuated mtDNA release into the cytosol in PGAM5-OV astrocytes (n = 6 per group, one-way ANOVA). Data are presented as means ± SD. *p < 0.05, **p < 0.01, ***p < 0.001.

**Figure S7. Ang2-si*Pgam5*-EVs exhibit a certain degree of stability both in vivo and in vitro.** **(A)** The number of si*Pgam5* copies in EVs was quantified by RT-qPCR at specified times following loading (n = 3 per group, one-way ANOVA). **(B-C)** Western blot analysis of PGAM5 protein expression to determine the effect of Ang2-si*Pgam5*-EVs injection following ICH (n = 6 per group, one-way ANOVA). Data are presented as means ± SD. *p < 0.05, **p < 0.01, ***p < 0.001.


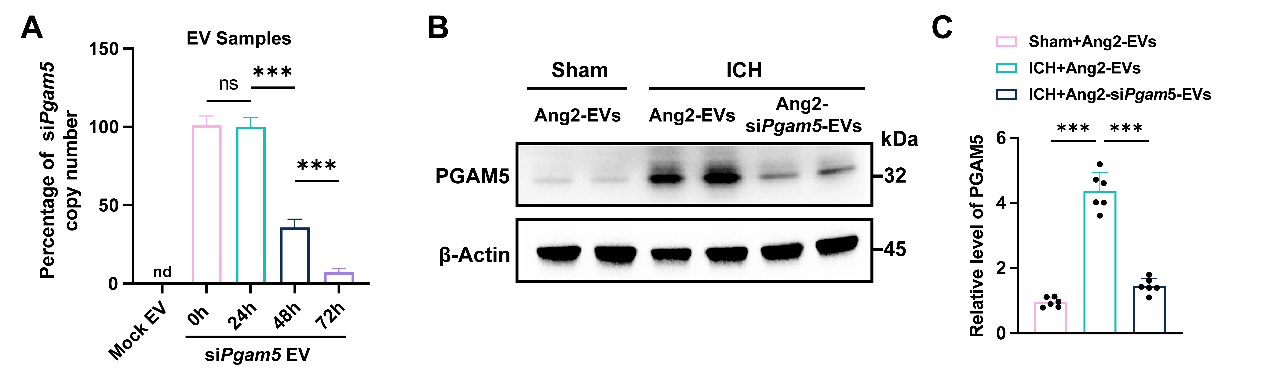


**Table S1. Sequences of siRNA in this study.**

| siRNA | Sequence |
| --- | --- |
| Control siRNA | 5'-UAAUGUAUUGGAACGGAU-3' |
| *Usp11* siRNA | (AC)CGATTCTATTGGCCTAGTA |
| *Pgam5* siRNA | Sence (5'-3'): CUGGAGAAGACGAGUUGACAUTT |
|  | Antisence (5'-3'): AUGUCAACUCGUCUUCUCCAGTT |

**Table S2. Sequences of primers used for PCR in this study.**

| Gene | Forward Primer(5'-3'） | Reverse Primer (5'-3'） |
| --- | --- | --- |
| *Ifnb*  （Mouse） | 5'-CCAGCTCCAAGAAAGGACGA-3' | 5'-TGGATGGCAAAGGCAGTGTA-3' |
| *Tnf*  （Mouse） | 5'-CCAAATGGCCTCCCTCTCAT -3' | 5'-TGGTGGTTTGCTACGACGTG-3' |
| *Il-6*  （Mouse） | 5'-CCAGAAACCGCTATGAAGTTCC-3' | 5'-CGGACTTGTGAAGTAGGGAAGG-3' |
| *ND1*  （Mouse） | 5'-CAAACACTTATTACAACCCAAGAACA-3' | 5'-TCATATTATGGCTATGGGTCAGG-3' |
| *ND2*  （Mouse） | 5'-CCATCAACTCAATCTCACTTCTATG-3' | 5'-GAATCCTGTTAGTGGTGGAAGG-3' |
| *L1 gDNA*  （Mouse） | 5'-TAGGAAATTAGTTTGAATAGGTGAGAGGGT-3' | 5'-TCCAGAAGCTGTCAGGTTCTCTGGC-3' |
| *Pgam5*  （Mouse） | 5'-ATCTGGAGAAGACGAGTTGACA-3' | 5'-CCTGTTCCCGACCTAATGGT-3' |
| *C3*  （Mouse） | 5'-AGATACTACGGAGGCGGCTA-3' | 5'-TTCTCAGCTATCCCGCAGTC-3' |
| *Serping1*  （Mouse） | 5'-AAGGTGGTGCTAAAATGCGG-3' | 5'-TCTCAGCGACCTCATGGGA-3' |
| *Psmb8*  （Mouse） | 5'-GGCAGAGACTTGCCCAAGAA-3' | 5'-GCAGGTCACTGACATCGGAA-3' |
| *Ptx3*  （Mouse） | 5'-CTCACAGAGCTCACACCACG-3' | 5'-CCGGTATCAGGAACCTCTGAC-3' |
| *S100a10*  （Mouse） | 5'-GATTCCACCCCTGGCTTCAA-3' | 5'-TGCAGCATCCCTGGCTTATC-3' |
| *Tgm1*  （Mouse） | 5'-TCTCTTACGGTGAGTGCAGC-3' | 5'-TCCCCTTGCCCACAGTTTTC-3' |
| *Pgam5*  （Human） | 5'-TCGTCCATTCGTCTATGACGC-3' | 5'-GGCTTCCAATGAGACACGG-3' |
| *Ccl5*  (Mouse) | 5'-GACACCACTCCCTGCTGCTT-3' | 5'-ACACTTGGCGGTTCCTTCG-3' |
| *Usp11*  (Mouse) | 5'-GCACCTTTCCTGGCTGTATC-3' | 5'-TCCCTGAGATGCCAGCTTAT-3' |
